# Apoplastic Venom Allergen-like Proteins of Cyst Nematodes Modulate the Activation of Basal Plant Innate Immunity by Cell Surface Receptors

**DOI:** 10.1371/journal.ppat.1004569

**Published:** 2014-12-11

**Authors:** Jose L. Lozano-Torres, Ruud H. P. Wilbers, Sonja Warmerdam, Anna Finkers-Tomczak, Amalia Diaz-Granados, Casper C. van Schaik, Johannes Helder, Jaap Bakker, Aska Goverse, Arjen Schots, Geert Smant

**Affiliations:** 1 Laboratory of Nematology, Wageningen University, Wageningen, The Netherlands; 2 Centre for BioSystems Genomics, Wageningen, The Netherlands; University of California, Riverside, United States of America

## Abstract

Despite causing considerable damage to host tissue during the onset of parasitism, nematodes establish remarkably persistent infections in both animals and plants. It is thought that an elaborate repertoire of effector proteins in nematode secretions suppresses damage-triggered immune responses of the host. However, the nature and mode of action of most immunomodulatory compounds in nematode secretions are not well understood. Here, we show that venom allergen-like proteins of plant-parasitic nematodes selectively suppress host immunity mediated by surface-localized immune receptors. Venom allergen-like proteins are uniquely conserved in secretions of all animal- and plant-parasitic nematodes studied to date, but their role during the onset of parasitism has thus far remained elusive. Knocking-down the expression of the venom allergen-like protein Gr-VAP1 severely hampered the infectivity of the potato cyst nematode *Globodera rostochiensis*. By contrast, heterologous expression of Gr-VAP1 and two other venom allergen-like proteins from the beet cyst nematode *Heterodera schachtii* in plants resulted in the loss of basal immunity to multiple unrelated pathogens. The modulation of basal immunity by ectopic venom allergen-like proteins in *Arabidopsis thaliana* involved extracellular protease-based host defenses and non-photochemical quenching in chloroplasts. Non-photochemical quenching regulates the initiation of the defense-related programmed cell death, the onset of which was commonly suppressed by venom allergen-like proteins from *G. rostochiensis*, *H. schachtii*, and the root-knot nematode *Meloidogyne incognita*. Surprisingly, these venom allergen-like proteins only affected the programmed cell death mediated by surface-localized immune receptors. Furthermore, the delivery of venom allergen-like proteins into host tissue coincides with the enzymatic breakdown of plant cell walls by migratory nematodes. We, therefore, conclude that parasitic nematodes most likely utilize venom allergen-like proteins to suppress the activation of defenses by immunogenic breakdown products in damaged host tissue.

## Author Summary

Plant-parasitic nematodes have a major impact on global food security, as they reduce the annual yield of food crops by approximately 10 percent. For decades, the application of non-selective toxic chemicals to infested soils controlled outbreaks of plant-parasitic nematodes. The recent bans on most of these chemicals has redirected attention towards a wider use of basal, broad-spectrum immunity to nematodes in crop cultivars. However, it is currently not known if this most ancient layer of immunity affects host invasion by plant-parasitic nematodes at all. Basal immunity in plants relies on the detection of molecular patterns uniquely associated with infections in the apoplast by surface-localized receptors. Here, we demonstrate that venom allergen-like proteins in secretions of soil-borne cyst nematodes suppress immune responses mediated by surface-localized pattern recognition receptors. Migratory stages of cyst nematodes most likely deliver venom allergen-like proteins together with a range of plant cell wall-degrading enzymes into the apoplast of host cells. We therefore conclude that these nematodes most likely secrete venom allergen-like proteins to modulate host responses triggered by the release of immunogenic fragments of damaged plant cell walls.

## Introduction

Soil-borne plant-parasitic nematodes are major constraints on global food security, as they reduce the annual yield of food crops by approximately 10 percent [1], [2]. This figure is a global average and may therefore be somewhat misleading. In areas where people depend on local cultivation of staple crops the effect of these microscopic roundworms can be devastating. The impact of plant-parasitic nematodes on food production provides plant breeders with a strong incentive to better exploit genetic variation in resistance to nematodes in crop cultivars. However, this requires knowledge of the mechanisms underlying the activation and suppression of plant innate immunity by plant-parasitic nematodes, an area which is currently underexplored [3], [4].

Plants utilize pattern recognition receptors belonging to the receptor-like kinase (RLK)/Pelle superfamily to detect extracellular microbes or their actions in the apoplast (i.e. the extracellular matrix; [5], [6]). The recognition of immunogenic microbe- and damage-associated molecular patterns by receptor-like kinases activates intracellular immune signaling pathways, resulting in a wide range of structural and chemical defenses [7], [8]. Several members of the RLK/Pelle superfamily in plants lack a cytoplasmic kinase domain, while they are nonetheless able to activate immune responses to pathogens (e.g. Cf-proteins in tomato; [9]–[11]). The activity of these so-called receptor-like proteins requires mediation by other transmembrane proteins, or cytoplasmic membrane-associated kinases, that function as co-factors within multimeric receptor complexes [12], [13]. At present, little evidence is available on the role of surface-localized pattern recognition receptors in immunity to parasitic nematodes in plants.

Recently, we showed that the receptor-like protein Cf-2 in tomato mediates dual disease resistance by guarding a common virulence target of a nematode and a fungus [14]. Perturbations of the apoplastic papain-like cysteine protease Rcr3^pim^ by two unrelated effectors from the leaf mold fungus *Cladosporium fulvum* and from the root parasitic nematode *Globodera rostochiensis* activate Cf-2-mediated disease resistance. The function of Rcr3^pim^, or any of its close homologs in tomato, has not yet been resolved [15]–[17]. Tomato plants harboring the Rcr3^pim^ allele, but not the receptor Cf-2, are far more susceptible to infections by *G. rostochiensis* than tomato plants lacking Rcr3^pim^ [14]. Apoplastic Rcr3^pim^ is a molecular target of the venom allergen-like protein Gr-VAP1 of *G. rostochiensis*, which is secreted by infective juveniles during the onset of parasitism. However, the role of this venom allergen-like protein (VAP), or its interaction with Rcr3^pim^, in nematode virulence is not clear.

Venom allergen-like proteins constitute a monophyletic clade of cysteine-rich secretory proteins within the Sperm Coating Protein/Tpx-1/Ag-5/Pr-1/Sc-7 (SCP/TAPS) superfamily ([18], [19]). Members of this clade show similarity to venom allergen 5 from vespid wasps, pathogenesis-related protein PR-1 from plants, brain tumor specific proteins in humans, and a wide range of other secreted proteins (reviewed in [18]). Venom allergen-like proteins have been identified in all animal- and plant-parasitic nematodes studied to date [19], [20]. They are even the most abundantly secreted proteins during the onset of parasitism of some animal-parasitic nematodes [19], [21]–[24]. In spite of their conservation, abundance, and strong association with the onset of parasitism, little is currently known of the function of venom allergen-like protein in nematode infections in plants and in animals [19].

Sedentary plant-parasitic nematodes, such as cyst nematodes (genera *Globodera* and *Heterodera*) and root-knot nematodes (genus *Meloidogyne*), deliver effectors into the apoplast and cytoplasm of host cells to induce the formation of a permanent feeding structure [25]–[27]. The permanent feeding structure is the sole source of plant nutrients for sedentary nematodes throughout their life [28]. Besides altering host cell metabolism and function, sedentary nematodes also use effectors to modulate host immunity [3], [27]. Specific immunity to nematodes in host plants often involves a programmed cell death in or around permanent feeding structures, resulting in the developmental arrest of feeding juveniles [3]. The most advanced sedentary nematodes deliver effectors into the cytoplasm of host cells to suppress the defense-related programmed cell death mediated by intracellular immune receptors [29], [30]. However, sedentary nematodes are extracellular parasites and their prolonged contact with surrounding host cells makes them also vulnerable to detection by surface-localized pattern recognition receptors [14]. Recent discoveries with the root-knot nematode *M. incognita* suggest that sedentary plant-parasitic nematodes may have adapted to this by evolving a separate set of apoplastic effectors to further control host immunity [31], [32].

The unique conservation of venom allergen-like proteins in secretions of animal- and plant-parasitic nematodes might point to a common activity of these effector proteins in the extracellular matrix of animal and plant cells. However, the composition, structure, and function of the extracellular matrix of animal and plant cells are fundamentally different [33], [34]. A possible exception might be that both in animals and plants nematodes encounter an innate immune system that relies on the surveillance of the extracellular matrix by surface-localized pattern recognition receptors [35], [36]. Earlier work has demonstrated that a secreted venom allergen-like protein from the animal-parasitic nematode *Necator americanus* acts in vitro as an antagonistic ligand of the integrin complement receptor 3, a pattern recognition receptor on the surface of human neutrophils [37]–[40]. This observation led us to investigate if venom allergen-like proteins of plant-parasitic nematodes similarly interfere with the functioning of surface-localized immune receptors in plants. To address this question, we first analyzed if venom allergen-like proteins are important for the onset of parasitism by silencing the expression of *Gr-VAP1* in infective juveniles of *G. rostochiensis*. Next, we analyzed the effect of ectopic venom allergen-like proteins in transgenic plants on susceptibility to nematodes, and diverse plant pathogenic fungi, oomycetes, and bacteria. Based on the response of these plants to the immunogenic epitope flg22 from bacterial flagellin [41], we concluded that the venom allergen-like proteins suppress basal plant defenses to biotic stresses. We further provide evidence that the breakdown of basal immunity by ectopic venom allergen-like proteins involves a plant cell wall-associated subtilisin-like serine protease, not previously linked to defense regulation in plants. Remarkably, our data also suggest that cyst nematodes exploit a trade-off mechanism between resistance to biotic and abiotic stress to ward off host defense responses.

## Results

### The venom allergen-like protein Gr-VAP1 is required for the onset of parasitism by *G. rostochiensis*

**Figure 1:**
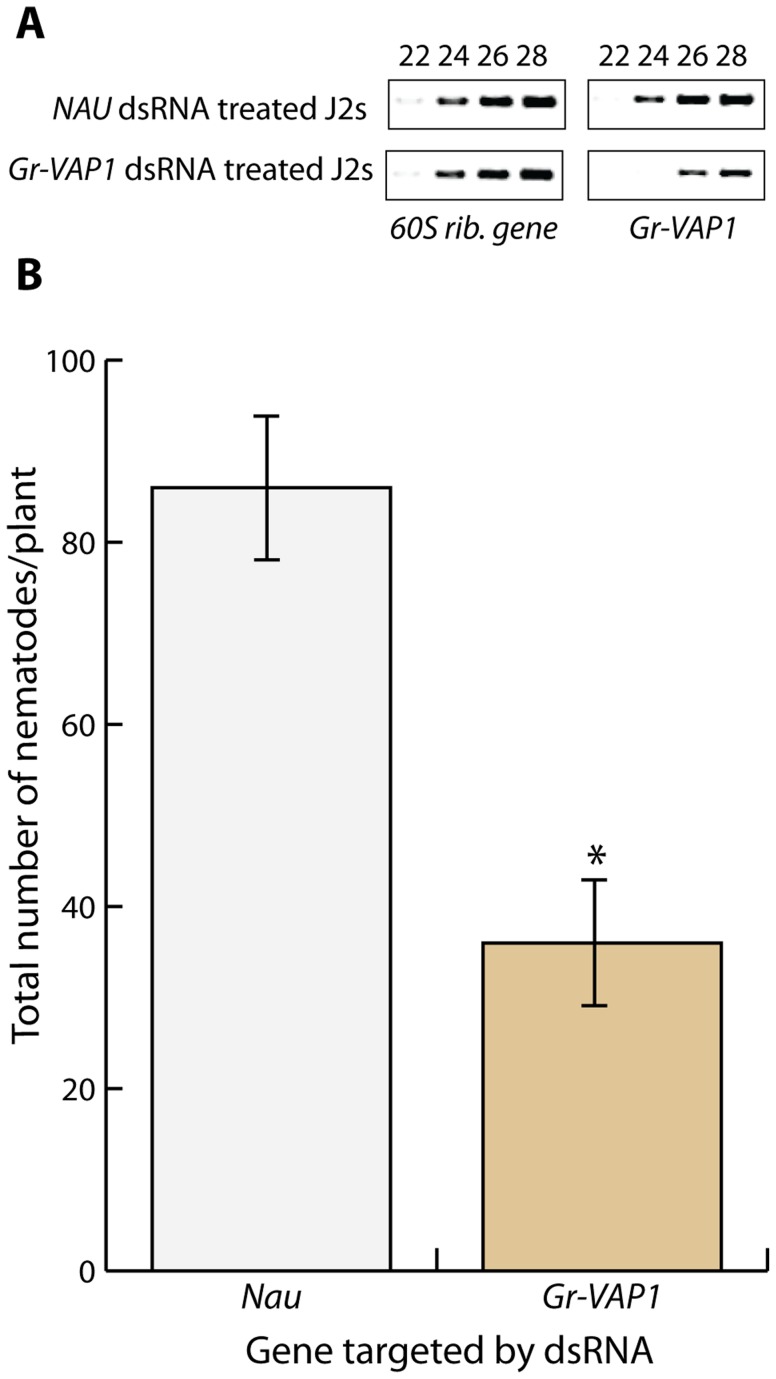
The venom allergen-like protein Gr-VAP1 is required for the onset of parasitism in host plants. (A) RNA interference specifically knocked down *Gr-VAP1* expression in pre-parasitic second stage juveniles of *G. rostochiensis*. Semi-quantitative reverse transcription-PCR of *Gr-VAP1* and a reference gene (*60S rib. gene*) in pre-parasitic second juveniles in double stranded RNA either matching the *Gr-VAP1* sequence or the sequence of the *NAU* gene of *Drosophila melanogaster* as control. Numbers indicate the cycles in the PCR. (B) The knockdown of *Gr-VAP1* expression significantly reduces the number of infective juveniles of *G. rostochiensis* inside roots of tomato plants (*S. lycopersicum*). Pre-parasitic second juveniles were either treated with double stranded RNA matching the *Gr-VAP1* or the *Nau* sequence. Bars represent standard error of mean of number of nematodes per plant at 7 days after inoculation over 10 replicates. Asterisk marks significance in a Student's t-test (with *P*-value <0.05).

To investigate whether venom allergen-like proteins are required for the onset of parasitism by sedentary plant-parasitic nematodes, we soaked infective juveniles of *G. rostochiensis* in double-stranded RNA, matching 820 base pairs of the *Gr-VAP1* transcript sequence. Reverse transcription PCR on nematodes, soaked in *Gr-VAP1*-specific dsRNA, showed a significant reduction in *Gr-VAP1* transcript levels, whereas the control treatment with dsRNA matching the *NAU* gene from *Drosophila melanogaster* did not alter *Gr-VAP1* expression (Fig. 1A). Next, susceptible tomato plants (*Solanum lycopersicum*, cultivar Moneymaker) were challenged with the dsRNA-treated infective juveniles, and monitored for nematode infections for 7 days post inoculation. Treatment with *Gr-VAP1*-specific dsRNA significantly reduced the number of nematodes inside tomato roots compared to the treatment with *NAU-*specific dsRNA (Fig. 1B). We therefore concluded that the apoplastic venom allergen-like protein Gr-VAP1 is required for the establishment of successful infections by *G. rostochiensis* during the onset of parasitism.

### *Gr-VAP1* expression coincides with nematode migration inside host plants

**Figure 2:**
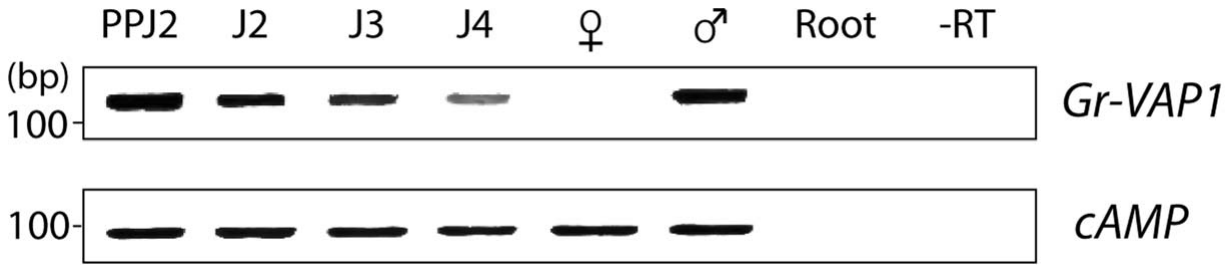
The expression of Gr-VAP1 coincides with host invasion and migration of *Globodera rostochiensis*. The expression of *Gr-VAP1*, as shown by semi-quantitative reverse transcription PCR, is highly up-regulated in the migratory stages of *G. rostochiensis* (ppJ2, J2, and males (♂)), while it declines after initiation of the permanent feeding site in the sedentary juvenile stages (J3 and J4, and adult females (♀). Changes in expression of *Gr-VAP1* were assessed using the constitutively expressed *cAMP-*dependent protein kinase (*cAMP*) gene in *G. rostochiensis* as reference. Reactions using uninfected tomato roots as template (Root) and without reverse transcriptase (-RT) were included as controls.

Both infective second-stage juveniles and adult males of *G. rostochiensis* migrate through host tissues, which causes significant damage to host cells. By contrast, intermediate juvenile stages and adult females are immobile, and thus induce little damage. To determine whether the expression of *Gr-VAP1* in *G. rostochiensis* coincides with either migration or sedentarism in host plants, we used semi-quantitative reverse transcription PCR on nematodes isolated from infected potato roots at different time points prior to and post host invasion (Fig. 2). *Gr-VAP1* was highly expressed in infective second stage juveniles during the onset of parasitism. Thereafter, the level of *Gr-VAP1* expression declined in successive sedentary juvenile stages inside host roots to total absence in sedentary adult females. However, the expression of *Gr-VAP1* was raised again in migratory adult males. We therefore concluded that the temporal expression of *Gr-VAP1* in *G. rostochiensis* coincides with nematode migration inside host plants.

### Ectopic Gr-VAP1 increases susceptibility of potato plants to *G. rostochiensis*

**Figure 3:**
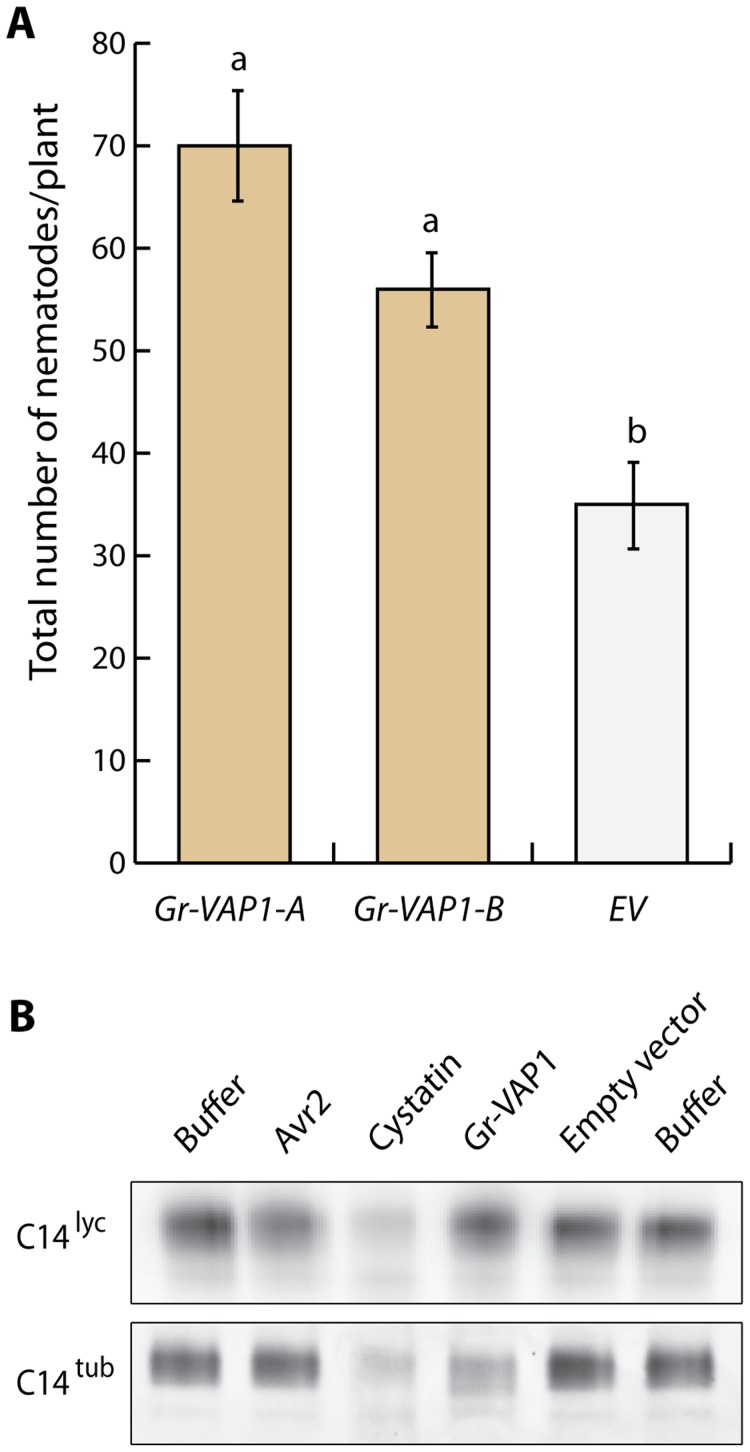
Apoplastic Gr-VAP1 suppresses immunity of potato plants to *G. rostochiensis*. (A) Transgenic potato plants stably overexpressing Gr-VAP1 in the apoplast show enhanced susceptibility to *G. rostochiensis*. The number of nematodes per plant was compared at 6 weeks post inoculation for two independent transgenic potato lines harboring either Gr-VAP1 (*Gr-VAP1-A* and *Gr-VAP1-B*) or the corresponding T-DNA insert of the empty binary expression vector (*EV*). The expression constructs included native signal peptide for secretion of Gr-VAP1. Bars represent standard errors of the means. Different letters indicate statistically significant differences between plant genotypes as determined with ANOVA (with *P*-values <0.05). (B) Apoplastic Gr-VAP1 perturbs the active site of the extracellular defense-related papain-like cysteine protease C14^tub^ of potato (*S. tuberosum*). Image shows binding of the fluorescent activity-based probe DCG-04 to the active site of C14^tub^ and C14^lyc^ of tomato (*S. lycopersicum*) following treatment with Gr-VAP1 isolated from apoplastic fluids of agroinfiltrated leaves. Treatments with the Avr2, egg white cystatin, and apoplastic fluids from agroinfiltrations with the empty binary expression vector (Empty vector), and with buffer alone (Buffer) were included as controls.

To examine whether Gr-VAP1 affects the susceptibility of host plants to *G. rostochiensis*, we generated transgenic potato plants ectopically overexpressing *Gr-VAP1*. Two randomly selected independent transgenic lines without any visible anomalies in shoots and roots were challenged with infective juveniles of *G. rostochiensis*. Six weeks after inoculation the number of adult females in plants expressing *Gr-VAP1* was significantly higher than in the corresponding empty vector control plants (Fig. 3A). To confirm that the altered nematode susceptibility correlates with *Gr-VAP1* expression, we used a real-time quantitative reverse transcription PCR on the two potato lines expressing *Gr-VAP1*. Transgenic line Gr-VAP1-A, that showed the highest nematode susceptibility, had a 7.9-fold higher expression of *Gr-VAP1* than transgenic line Gr-VAP1-B. We therefore concluded that ectopic Gr-VAP1 enhances the susceptibility of potato plants to *G. rostochiensis.*

Like the effector Avr2 from *C. fulvum* [42], we expected Gr-VAP1 to interact with other extracellular papain-like cysteine proteases in different host plant species of *G. rostochiensis*. We used DCG-04 activity profiling to demonstrate that Gr-VAP1 also perturbs the active site of the apoplastic papain-like cysteine protease C14^tub^ from potato (*S. tuberosum*) (Fig. 3B). By contrast, Gr-VAP1 does not interfere with the binding of fluorescent DCG-04 to apoplastic C14^lyc^ from tomato (*S. lycopersicum*). For this experiment, Gr-VAP1 and C14^tub/lyc^ were separately produced in the apoplast of agroinfiltrated leaves of *Nicotiana benthamiana*. Protease activity was subsequently determined by the binding of fluorescent DCG-04 to C14^tub^ and C14^lyc^ in the presence of Gr-VAP1 on gels of mixtures of isolated apoplastic fluids. The experiment was repeated three times, and each attempt resulted in significantly less binding of the fluorescent probe to C14^tub^, but not to C14^lyc^ (S1 Figure). Only C14^tub^ from potato is under strong diversifying selection, because of which it is thought to be involved defenses [43]. We therefore concluded that the enhanced susceptibility by ectopic Gr-VAP1 in potato most likely involves the perturbation of C14^tub^ and perhaps other apoplastic papain-like cysteine proteases.

### Ectopic venom allergen-like proteins enhance the susceptibility of *Arabidopsis* to multiple unrelated plant pathogens

*Arabidopsis thaliana* is a far better model to study the molecular changes induced by venom allergen-like proteins in plants than either potato or tomato. However, *G. rostochiensis* is not able to establish infections in *A. thaliana*. We therefore continued our investigations with two homologous venom allergen-like proteins from the beet cyst nematode *Heterodera schachtii*, which is a parasite of *A. thaliana*. These two venom allergen-like proteins are formally designated as Nem-*Hsc*-SCP/TAPS-1A and Nem-*Hsc*-SCP/TAPS-2A [18], but for the remainder of this paper they are referred to as Hs-VAP1 and Hs-VAP2. Hs-VAP1 is 81.4 percent identical to Gr-VAP1, while Hs-VAP2 shows only 34.8 percent sequence identity to Gr-VAP1. In comparison, a previously reported venom allergen-like protein from the root-knot nematode *Meloidogyne incognita* (hereafter named Mi-VAP1; [44]) shows about 28.6% identity to Gr-VAP1 and Hs-VAP1, while it is for 33.9% of its sequence identical to Hs-VAP2 (S2 Figure).

**Figure 4:**
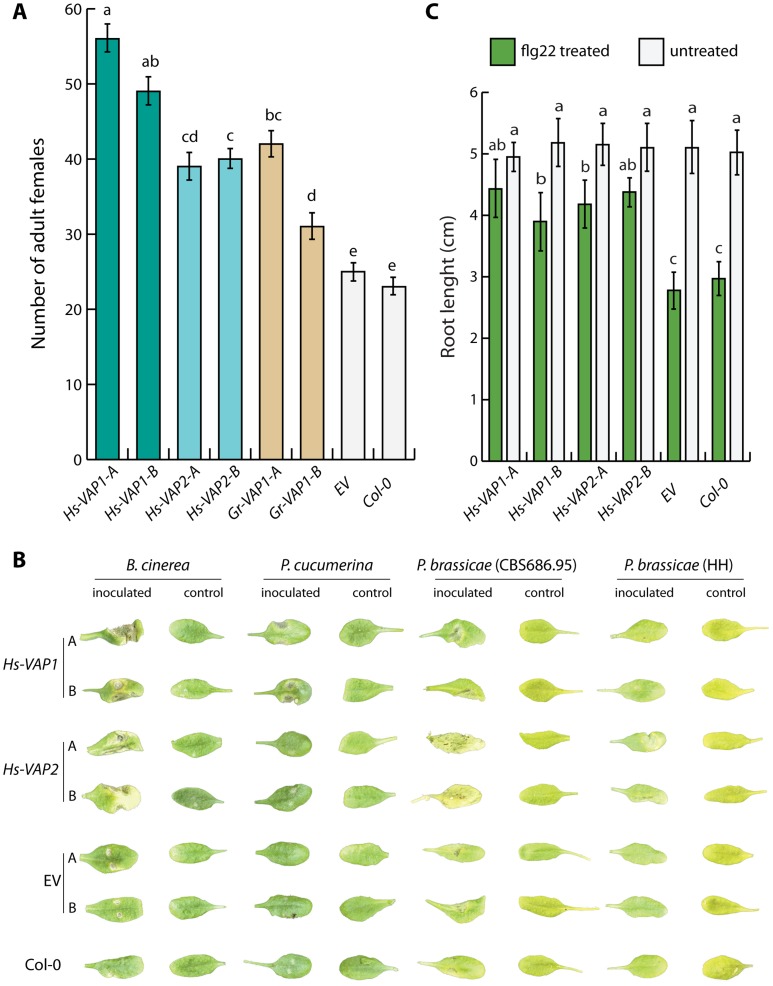
Ectopic venom allergen-like proteins suppress basal immunity in *Arabidopsis thaliana*. (A) Heterologous expression of the venom allergen-like protein Gr-VAP1 from *G. rostochiensis*, and Hs-VAP1 and Hs-VAP2 from *Heterodera schachtii* in the apoplast of transgenic Arabidopsis lines enhances their susceptibility to *H. schachtii*. Two independent transgenic lines per construct (-A and -B) were compared with corresponding transgenic line harboring the T-DNA of the empty vector (EV) and wild type *A. thaliana* (Col-0). Bars represent mean number of nematodes per plants with standard errors of the means. Letters indicate statistical significance when using *P*-value <0.05 as threshold. (B) Ectopic Hs-VAP1 and Hs-VAP2 enhance development of disease symptoms of fungal and oomycete pathogens in leaves of transgenic Arabidopsis lines. Pictures show symptoms on leaves inoculated with *Botrytis cinerea*, *Plectosphaerella cucumeria*, and two isolates of *Phytophthora brassicae*, or mock inoculated. (C) Ectopic Hs-VAP1 and Hs-VAP2 suppress seedling growth response of Arabidopsis to the immunogenic peptide flg22. Bars represent mean root length of transgenic lines with standard error of mean after 10 days in the presence or absence of 10 µM flg22.

To investigate if ectopic venom allergen-like proteins from *H. schachtii* and *G. rostochiensis* alter the susceptibility of *A. thaliana* to cyst nematodes, we generated transgenic plants overexpressing *Gr-VAP1*, *Hs-VAP1*, and *Hs-VAP2*, including their native signal peptides for secretion. We challenged two independent single insertion lines, without visible anomalies in shoots and roots, of each construct with infective juveniles of *H. schachtii*. Twenty-eight days after inoculation the number of females per plant in plants expressing *Gr-VAP1*, *Hs-VAP1*, and *Hs-VAP2* was significantly higher than in the corresponding transgenic empty vector line or in the wild type Col-0 plants (Fig. 4A). We therefore concluded that venom allergen-like proteins from two unrelated cyst nematodes commonly enhance the susceptibility of different plant species to nematode infections.

Next, we reasoned that if the VAP-enhanced susceptibility of the transgenic Arabidopsis lines to cyst nematodes involves modulation of the innate immunity, these lines might also be more susceptible to entirely unrelated plant pathogens. To test this, we analyzed the transgenic Arabidopsis lines overexpressing *Hs-VAP1* and *Hs-VAP2* for their susceptibility towards *Botrytis cinerea*, *Plectosphaerella cucumerina*, a virulent and a non-virulent isolate of *Phytophthora brassicae*, *Alternaria brassicicola*, *Verticillium dahliae*, and *Pseudomonas syringae* pv. *tomato* (Fig. 4B, and S3 Figure). The overexpression of both *Hs-VAP1* and *Hs-VAP2* significantly increased the severity of the grey mold symptoms caused by *B. cinerea* in the transgenic Arabidopsis plants (S4A Figure). Similarly, both *Hs-VAP1* and *Hs-VAP2* significantly increased susceptibility of Arabidopsis plants to infections by *P. syringae* pv. *tomato* (S3A Figure). Only Arabidopsis plants overexpressing *Hs-VAP1* showed larger necrotic lesions following the inoculation with the fungus *P. cucumerina* (S4B Figure). By contrast, the oomycete *P. brassicae* (isolate CBS686.95) only caused faster developing and larger lesions on transgenic Arabidopsis expressing *Hs-VAP2* (S4C Figure). Surprisingly, the *P. brassicae* isolate HH, which is not virulent on wild type *A. thaliana* Col-0, was able to colonize transgenic *A. thaliana* lines expressing *Hs-VAP2* (S4D Figure). However, neither *Hs-VAP1* nor *Hs-VAP2* altered the susceptibility of *A. thaliana* towards the fungal pathogens *A. brassicicola* or *V. dahliae*, both of which do not cause expanding lesions in Arabidopsis ecotype Col-0. Altogether, our data suggests that ectopic Hs-VAP1 and Hs-VAP2, albeit differently, modulate basal innate immunity of *A. thaliana* toward multiple, but not all, plant pathogens. Furthermore, ectopic VAPs specifically altered the susceptibility of Arabidopsis to pathogenic microbes that typically cause expanding lesions in their necrotrophic phase (i.e. *B. cinerea*, *P. cucumerina*, *P. brassicae*, and *P. syringae* pv. *tomato*).

### Ectopic venom allergen-like proteins abrogate the response of Arabidopsis to the immunogenic peptide flg22

The flagella of *P. syringae* pv. *tomato* harbor an immunogenic epitope of twenty two amino acids (flg22) that is recognized as a pathogen-associated molecular pattern in Arabidopsis [45]. Prolonged exposure to flg22 elicits a persistent basal immune response in seedlings of Arabidopsis Col-0 plants, which occurs at the expense of plant growth [45]. We used this phenotype to confirm that ectopic venom allergen-like proteins undermine basal immunity in our transgenic Arabidopsis lines. As expected, treatment with flg22 significantly inhibited seedling growth in both wild-type Arabidopsis and in our transgenic lines harboring the empty expression vector (Fig. 4C). By contrast, the expression of *Hs-VAP1* and *Hs-VAP2* in Arabidopsis seedlings largely abrogated this growth inhibition by flg22 (Fig 4C; S5 Figure). Remarkably, the leaves of the transgenic plants overexpressing *Hs-VAP1* and *Hs-VAP2* also remained much greener as compared to the leaves of wild type Col-0 and empty vector control plants following the treatment with flg22. As the perception of flg22 in Arabidopsis is mediated by the extracellular pattern recognition receptor FLS2 [45], we concluded that ectopic venom allergen-like proteins most likely modulate the activation of basal immunity by surface-localized immune receptors.

### Extracellular papain-like cysteine proteases regulate immunity to cyst nematodes in Arabidopsis

**Figure 5:**
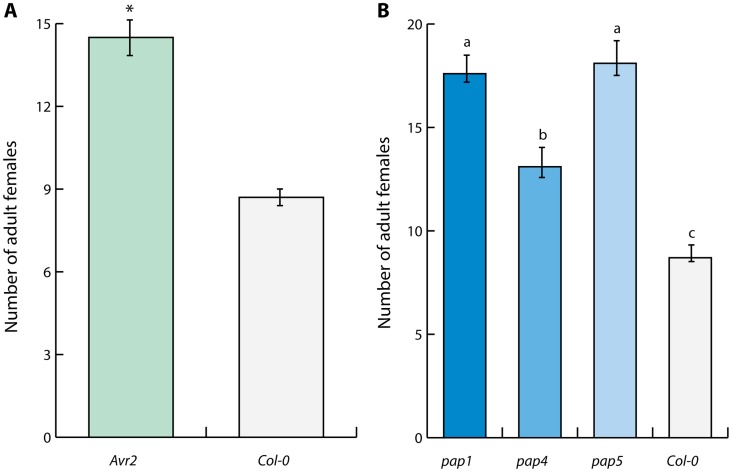
Defense-related extracellular papain-like cysteine proteases regulate immunity to cyst nematodes in Arabidopsis. (A) Inhibition of papain-like cysteine proteases by heterologous expression of the apoplastic effector Avr2 from *Cladosporium fulvum* in transgenic Arabidopsis lines suppresses immunity to *H. schachtii*. The bars represent mean number of nematodes per plant with standard error at four weeks post inoculation in transgenic line harboring ectopic Avr2 in the apoplast (Avr2) and the corresponding wild type Arabidopsis. Asterisk indicates statistical significance when using *P*-value <0.05 as threshold (Student's t-test). (B) Members of the extracellular papain-like cysteine protease family AtPAP in Arabidopsis are required for immunity to *H. schachtii*. Bars represent mean number of nematodes per plant with standard error of mean. Different letters indicate statistically significant differences between homozygous knockout mutants *pap1*, *pap4*, *pap5* and corresponding wild type Arabidopsis at four weeks after inoculation determined with ANOVA (with *P*-values <0.05).

The apoplastic effectors Avr2 of *C. fulvum* and Gr-VAP1 of *G. rostochiensis* commonly inhibit the extracellular papain-like protease Rcr3^pim^ in tomato [14]. Although *C. fulvum* is not a pathogen of Arabidopsis either, ectopic Avr2 has been shown to interact with multiple extracellular papain-like cysteine proteases of Arabidopsis [42]. To investigate if heterologous expression of Avr2 through its interactions with extracellular papain-like cysteine proteases also affects susceptibility of Arabidopsis to nematode infections, we challenged transgenic Arabidopsis plants stably overexpressing Avr2 with *H. schachtii*. Four weeks post inoculation the number of adult females of *H. schachtii* per root was significantly higher in plants overexpressing Avr2 than in the corresponding wild type Arabidopsis plants (Fig. 5A). This data shows that the inhibition of extracellular papain-like cysteine proteases by promiscuous effectors from different non-adapted plant attackers (i.e. Gr-VAP1 and Avr2) undermines basal immunity in Arabidopsis.

To further confirm the importance of extracellular papain-like cysteine proteases in basal immunity to nematode infections, we challenged the homozygous knockout mutants *pap-1*, *pap-4*, and *pap-5* of Arabidopsis with *H. schachtii*. Members of the *pap* gene family are the closest homologs of Rcr3^pim^ in Arabidopsis [14]. The loss of functional *pap* genes in all three mutant Arabidopsis lines resulted in significantly enhanced susceptibility to *H. schachtii* (Fig. 5B). We therefore conclude that conserved extracellular protease-based immune signaling networks most likely regulate basal immunity to plant-parasitic nematodes in multiple unrelated plants.

### Ectopic venom allergen-like proteins regulate apoplastic and chloroplastic immune signaling in Arabidopsis

To better understand the molecular basis of the suppression of basal immunity by venom allergen-like proteins, we analyzed the transcriptomes of Arabidopsis lines expressing *Hs-VAP1*, *Hs-VAP2*, and the corresponding transgenic empty vector control plants. In total, the expression of 1294 genes was significantly down-regulated, while 535 genes were significantly up-regulated in the Arabidopsis lines overexpressing either *Hs-VAP1* or *Hs-VAP2* (False Discovery Rate <0.05) (S6A Figure and S6B Figure). More than sixty percent of the Arabidopsis genes strongly down-regulated by ectopic *Hs-VAP1* and *Hs-VAP2* encode a protein that is either predicted to be extracellular or localized to the plasma membrane (S4C Figure; S1 Table and S2 Table). By contrast, the predicted subcellular location of the products of the genes strongly up-regulated by either Hs-VAP1 or Hs-VAP2 are more evenly distributed over different cellular compartments (S3 Table and S4 Figure).

To resolve specific pathways particularly affected by the overexpression of *Hs-VAP1* and *Hs-VAP2* in *A. thaliana*, we subjected all differentially expressed genes to a KEGG pathway gene set enrichment analysis [46], [47]. The KEGG pathway most significantly altered by both Hs-VAP1 and Hs-VAP2 in Arabidopsis is named ‘Plant-pathogen interactions' (KO04626; S5 Table; FDR <10^-12^). The vast majority of these Arabidopsis genes currently assigned to this pathway have been associated with innate immunity to plant pathogens [48]. We therefore concluded that the overexpression of venom allergen-like proteins in Arabidopsis particularly affects molecular components in immune signaling pathways.

To further investigate the expression of specific genes associated with the loss of immunity in the transgenic Arabidopsis plants overexpressing *Hs-VAP1* and *Hs-VAP2*, we first focused on the most down-regulated genes (Table 1). The annotations of many of the most down-regulated genes point to an involvement of plant cell wall-associated processes such as modification (e.g. plant invertase/pectin methylesterase inhibitor family, and glycosyl hydrolases), signaling (e.g. proline-rich extension-like receptor kinases), and protein processing (e.g. subtilisin-like serine proteases).

**Table 1:** Ten most down-regulated transcripts by ectopic venom allergen-like proteins in Arabidopsis.

Transgene	Gene model	Name	Function	Cellular Location	FC*	P-value*	FDR*
**Hs-VAP1**	AT4G21630.1	Subtilase family protein (Sbt3.14)	Serine-type endopeptidase	Cell wall	−32.0	1.75381E-36	4.07956E-33
	AT3G01345.1	Glycoside hydrolase family 35	Beta-galactosidase	Mitochondrion	−9.5	7.8436E-215	1.6421E-210
	AT5G49180.1	Pectin methylesterase	Cell wall modification	Cell wall	−5.7	3.97036E-21	1.1707E-18
	AT3G14520.1	Terpenoid cyclase	Terpene synthase activity	Cytosol	−4.5	2.74283E-16	4.41702E-14
	AT2G02490.1	Unknown protein	-	-	−4.2	5.34408E-19	1.18673E-16
	AT5G20330.1	Beta-1,3-glucanase 4 (BETAG4)	Hydrolase activity	Extracellular	−4.0	1.53528E-18	3.18228E-16
	AT1G10620.1	Proline-rich extension-like receptor kinase 11 (PERK11)	Receptor-like kinase	Plasma membrane	−4.0	2.14972E-14	2.92237E-12
	AT2G34870.1	Maternal effect embryo arrest 26 (MEE26)	Unknown	Mitochondrion	−4.0	1.51571E-48	6.34628E-45
	AT2G36325.1	GDSL-like Lipase/Acylhydrolase superfamily protein	Hydrolase activity	Extracellular	−4.0	1.25176E-11	1.38654E-09
	AT5G20390.1	Glycoside hydrolase super family 17	Hydrolase activity	Extracellular	−3.9	1.11178E-27	7.05308E-25
**Hs-VAP2**	AT3G01345.1	Glycoside hydrolase family 35	Beta-galactosidase	Mitochondrion	−9.5	3.3601E-183	7.0198E-179
	AT2G02490.1	Unknown protein	-	-	−4.2	8.47065E-22	1.17979E-18
	AT5G24240.1	Phosphatidylinositol 3- and 4-kinase	Phosphotransferase	Chloroplast, peroxisome	−4.3	5.7319E-68	3.99169E-64
	AT5G40348.2	Unknown	-	-	−4.0	9.87293E-13	5.75069E-10
	AT1G35310.1	MLP-like protein 28 (MLP28)	Unknown, defense	Nucleus	−3.7	1.92458E-23	3.09296E-20
	AT4G12890.1	Gamma interferon responsive lysosomal thiol reductase (GILT)	Oxidoreductase	Extracellular	−3.7	3.43315E-13	2.24142E-10
	AT4G21630.1	Subtilase family protein (Sbt3.14)	Serine-type endopeptidase	Cell wall	−3.6	1.34797E-21	1.76011E-18
	AT2G34870.1	Maternal effect embryo arrest 26 (MEE26)	Unknown	Mitochondrion	−3.4	1.07604E-36	3.74676E-33
	AT4G12960.1	Gamma interferon responsive lysosomal thiol reductase (GILT)	Oxidoreductase	Extracellular	−3.1	3.24747E-27	6.16783E-24
	AT1G67626.1	Unknown	-	-	−3.1	1.24615E-09	4.00531E-07

Differentially expressed genes in transgenic *Arabidopsis thaliana* overexpressing *Hs-VAP1* and *Hs-VAP2* relative to the corresponding transgenic empty vector control plants (for full lists see S1 Table and S2 Table).

* The fold change (FC) is calculated as standardized log2-transformed counts per million relative to transgenic plants harboring the corresponding empty vector.

**Figure 6:**
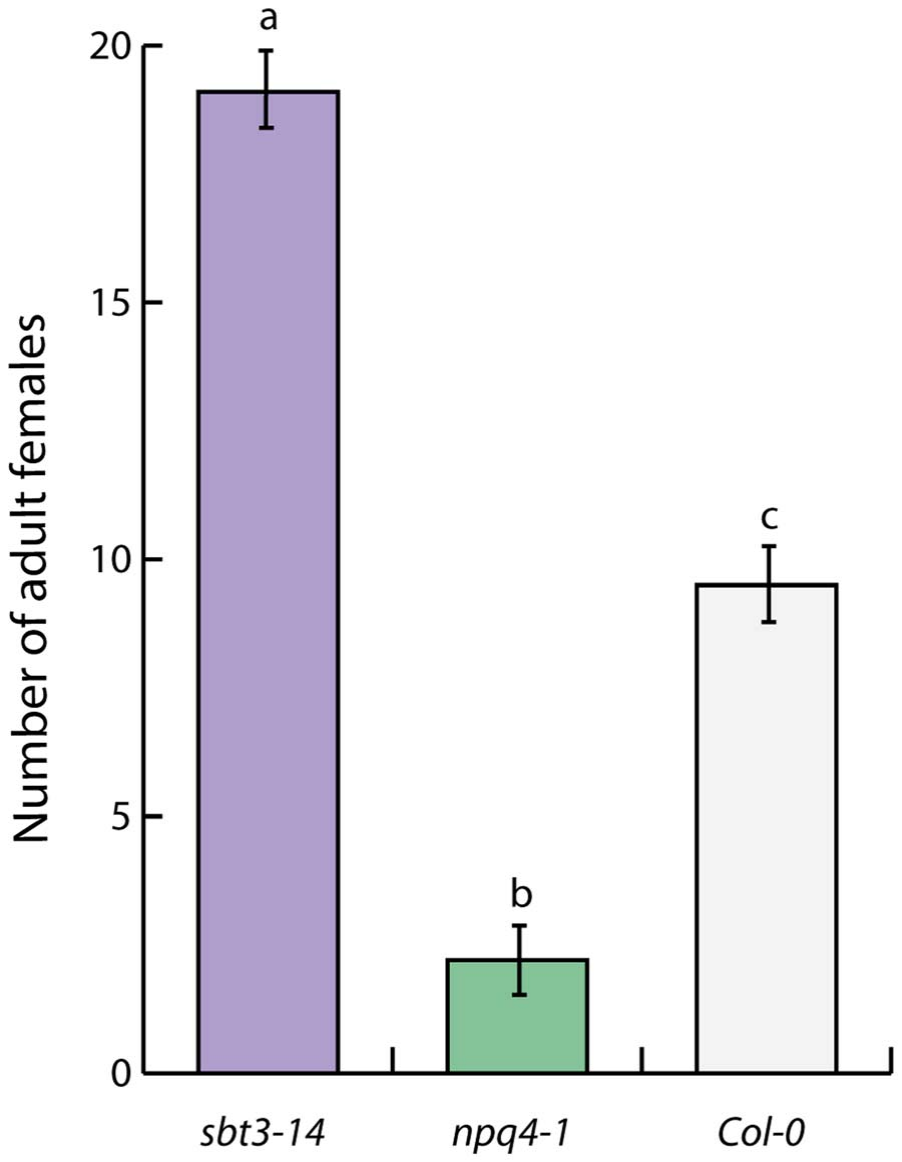
A plant cell wall-associated subtilase and non-photochemical quenching in chloroplasts regulate immunity to plant-parasitic nematodes. The lack of the subtilisin-like serine protease AtSBT3.13 and the chlorophyll-associated Photosystem II subunit S protein in homozygous Arabidopsis mutants (*sbt3.14* and *npq4-1*, respectively) significantly alters their susceptibility to *H. schachtii*. Bars represent mean number of nematodes per plant with standard error of mean. Different letters indicate statistically significant differences between homozygous knockout mutants and corresponding wild type Arabidopsis at four weeks after inoculation (determined with ANOVA (with *P*-values <0.05).

An exceptionally strong down-regulation was observed for gene locus AT4G21630 in *Hs-VAP1*-overexpressing Arabidopsis plants (Log2 fold change  = −32.0). Albeit less, AT4G21630 was also strongly down-regulated in the transgenic Arabidopsis lines overexpressing *Hs-VAP2*. AT4G21630 encodes a putative plant cell wall-associated subtilase-like serine protease (i.e. AtSBT3.14; [49]). The role of AtSBT3.14 in Arabidopsis is not known, but a closely related homolog from the same subtilase subfamily, named AtSBT3.3, acts as an extracellular molecular switch in priming of defense responses [50]. To investigate if AtSTB3.14 is required for basal immunity of Arabidopsis to the cyst nematodes, we challenged a homozygous knockout mutant line with *H. schachtii*. Four weeks post inoculation the number of adult females was almost twice as high in the *Atsbt3.14* knock-mutant, as compared to the corresponding Col-0 wild type Arabidopsis plants (Fig. 6). We therefore concluded that the strong down-regulation of *AtSBT3.14* most likely contributes to the loss of basal immunity in Arabidopsis plants overexpressing *Hs-VAP1* and *Hs-VAP2*.

Next, we focused on four of the most up-regulated transcripts in the transgenic Arabidopsis plant overexpressing *Hs-VAP1* and *Hs-VAP2* (Log2 fold change>30.9; Table 2). Two of these transcripts derive from gene loci encoding unknown proteins (i.e. AT1G44608 and AT1G44542). However, the two other transcripts are splice variants from the same and most up-regulated gene in both *Hs-VAP1* and *Hs-VAP2* overexpressing plants (i.e. AT1G44575, or *NPQ4*). *NPQ4* encodes the chlorophyll-associated Photosystem II subunit S protein (PsbS), which is involved in non-photochemical quenching of excess excitation energy [51].

**Table 2:** Ten most up-regulated transcripts by ectopic venom allergen-like proteins in Arabidopsis.

Transgene	Gene model	Name	Function	Cellular Location	FC*	*P*-value	FDR
**Hs-VAP1**	AT1G44575.1	Photosystem II subunit S (PsbS)	Nonphotochemical quenching	Chloroplast	36.1	1.9179E-141	2.0076E-137
	AT1G44608.1	Unknown protein	-	-	33.2	1.2297E-60	6.43594E-57
	AT1G44542.1	Cyclase family protein	Unknown	-	32.6	3.0409E-47	1.06102E-43
	AT1G44575.2	Photosystem II subunit S (PsbS)	Nonphotochemical quenching	Chloroplast	30.9	1.42748E-20	3.60053E-18
	AT4G19690.1	Iron regulated transport protein 1 (IRT1)	Metal ion transport	Plasma membrane	5.7	5.63033E-34	7.36693E-31
	AT5G48000.1	Cytochrome P450 family 780 polypeptide A2 (CYP708 A2)	Oxygen binding	Chloroplast	3.1	1.80855E-10	1.78594E-08
	AT3G16430.1	Jacalin-related lectin (31)	Copper ion binding	Extracellular	2.2	1.09992E-07	7.6248E-06
	AT2G36260.1	Iron-sulfur cluster biosynthesis family protein	Ion-sulfur cluster binding	Mitochondrion	2.1	2.2514E-09	1.87781E-07
	AT5G36270.1	Pseudogene of dehydroascorbate reductase (DHAR)	Redox homeostasis	Chloroplast	2.0	2.33035E-05	0.0010
	AT2G27420.1	Papain-like cysteine protease 4 (PAP4)	Proteolysis	Extracellular	2.0	4.78317E-06	0.0002
**Hs-VAP2**	AT1G44575.1	Photosystem II subunit S (PsbS)	Nonphotochemical quenching	Chloroplast	35.8	1.5741E-125	1.6443E-121
	AT1G44608.1	Unknown protein	-	-	33.3	7.93594E-60	4.14494E-56
	AT1G44542.1	Cyclase family protein	Unknown	-	32.7	4.47837E-48	1.87124E-44
	AT1G44575.2	Photosystem II subunit S (PsbS)	Nonphotochemical quenching	Chloroplast	30.7	1.82146E-18	1.9027E-15
	AT4G19690.1	Iron regulated transport protein 1 (IRT1)	Metal ion transport	Plasma membrane	5.7	5.8572E-32	1.74812E-28
	AT5G48000.1	Cytochrome P450 family 780 polypeptide A2 (CYP708 A2)	Oxygen binding	Chloroplast	2.7	3.37794E-07	4.35629E-05
	AT5G28020.6	Cysteine synthase (Cys D2)	Cysteine biosynthesis	Mitochondrion	2.5	5.14708E-08	8.672E-06
	AT2G36260.1	Iron-sulfur cluster biosynthesis family protein	Ion-sulfur cluster binding	Mitochondrion	2.5	1.35842E-12	7.6703E-10
	AT5G35935.1	Copia-like retrotransposon family	Retrotransposon	-	2.3	9.60494E-18	9.1212E-15
	AT5G36270.1	Pseudogene of dehydroascorbate reductase (DHAR)	Redox homeostasis	Chloroplast	2.2	7.03342E-06	0.0005

Differentially expressed genes in transgenic *Arabidopsis thaliana* overexpressing *Hs-VAP1* and *Hs-VAP2* relative to the corresponding transgenic empty vector control plants (for full lists see S3 Table and S4 Table).

* The fold change (FC) is calculated as standardized log2-transformed counts per million relative to transgenic plants harboring the corresponding empty vector.

Recently, it was shown that the Arabidopsis knockout mutant *npq4-1* lacking PsbS displays an enhanced response to flg22 [52]. The *npq4-1* knockout mutant is also less attractive to herbivorous insects than the corresponding wild type Arabidopsis plants [53]. We used the *npq4-1* knockout mutant to demonstrate that the lack of PsbS enhances immunity of Arabidopsis to *H. schachtii* (Fig. 6). We therefore conclude that the constitutively enhanced expression of *NPQ4* by ectopic *Hs-VAP1* and *Hs-VAP2* in transgenic Arabidopsis most likely undermines their ability to mount an adequate immune response.

### Apoplastic venom allergen-like proteins suppress defense-related programmed cell death activated by cell surface receptors

**Figure 7:**
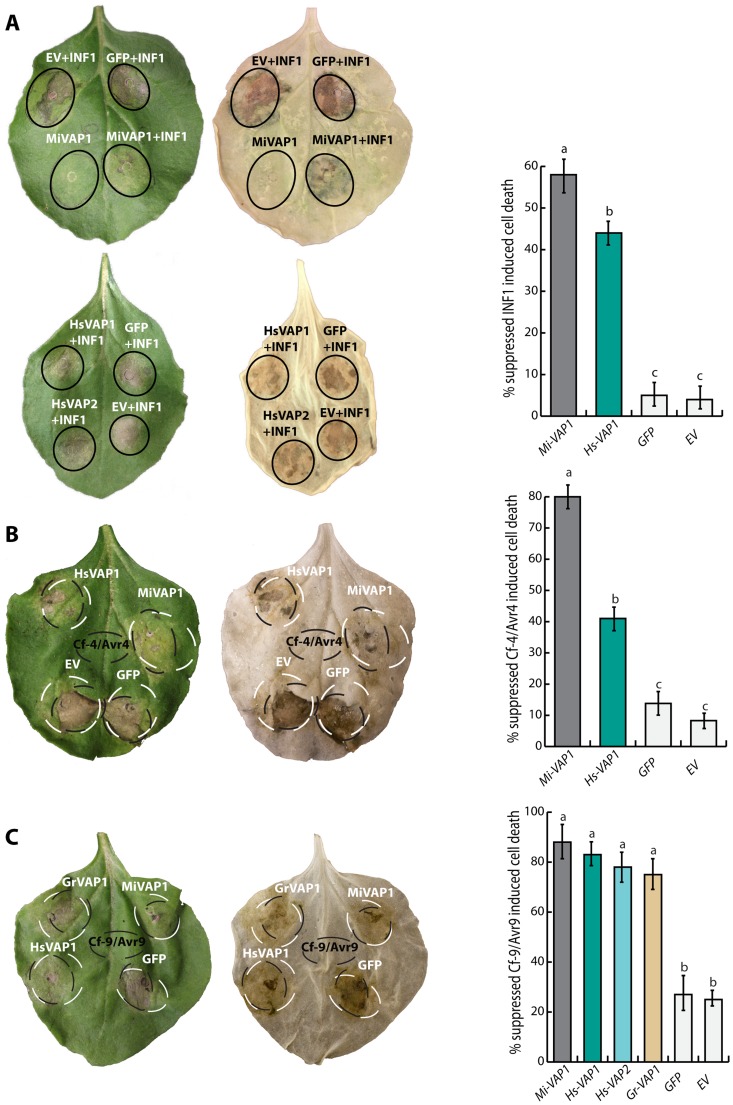
Ectopic venom allergen-like proteins from cyst and root-knot nematodes selectively suppress defense-related programmed cell death. (A) Agroinfiltration assays in *Nicotiana benthamiana* showing the transient co-expression in the apoplast of cell death inducing elicitin *INF1* of *Phytophthora infestans* and venom allergen-like proteins from *G. rostochiensis* (Gr-VAP1), *H. schachtii* (Hs-VAP1 and Hs-VAP2), and *Meloidogyne incognita* (Mi-VAP1). Co-expressions with the corresponding empty binary vector (EV) and green fluorescent protein (GFP) were included as controls. (B and C) Transient co-expression of receptor-like proteins Cf-4 and Cf-9 from tomato and their cognate elicitors Avr4 and Avr9 from *C. fulvum* with venom allergen-like proteins and controls as described above. Photographs were taken 4 days post infiltration for INF1, and 7 days post infiltration for Cf-4/Avr4 and Cf9/Avr9. The bars represent the mean number of events in which cell death suppression was observed for a total of 60 inoculation spots over 5 biological replicates (with standard error of mean). Different letters indicate a significant difference when using *P*-value <0.05 (in an ANOVA).

The increase of non-photochemical quenching capacity may block the activation of singlet oxygen-dependent programmed cell death [54], [55]. To investigate if the venom allergen-like proteins are able to suppress programmed cell death, we transiently co-expressed several inducers of cell death and nematode VAPs in leaves of *Nicotiana benthamiana* (S6 Table; Fig. 7). Because *N. benthamiana* is not a host of *G. rostochiensis* or *H. schachtii*, we also included the venom allergen-like protein Mi-VAP1 from the polyphagous *M. incognita* in these cell death suppression assays. Both Mi-VAP1 and Hs-VAP1 consistently suppressed the cell death induced by the *Phytophthora infestans* elicitin INF1 (Fig. 7A). Both Mi-VAP1 and Hs-VAP1 also suppressed the cell death induced by extracellular receptor protein Cf-4 from tomato and its cognate elicitor Avr4 from *C. fulvum* (Fig. 7B). All tested VAPs similarly suppressed the cell death induced by the extracellular receptor-like protein Cf-9 from tomato and its cognate elicitor Avr9 from *C. fulvum* (Fig. 7C). Surprisingly, none of the venom allergen-like proteins suppressed the cell death responses induced by several cytoplasmic immune receptors and their cognate elicitors (e.g. Rx1, Gpa2, R3a, Blb2). To confirm that the ectopic VAPs harboring their native signal peptide for secretion are indeed secreted to the apoplast in planta, we analyzed apoplastic fluids isolated from agroinfiltrated leaf of *N. benthamiana* on western blots using antiserum towards an additional carboxyl terminal FLAG affinity tag on the proteins (S7 Figure). Taken together, we conclude that apoplastic venom allergen-like proteins selectively suppress the activation of the programmed cell death by surface-localized immune receptors.

## Discussion

Since their first identification in the canine hookworm *Ancylostoma caninum* (Ac-ASP; [21]) and the root-knot nematode *M. incognita* (Mi-MSP1/VAP1; [44]) venom allergen-like proteins are thought to be crucial for the onset of parasitism of nematodes in animals and plants. However, in spite of their conservation, relative abundance in nematode secretions, and strong association with the onset of parasitism, the role of venom allergen-like proteins in host-parasite interactions has so far remained elusive [18], [19]. To date, the only available functional data on the role of a venom allergen-like protein in secretions of parasitic nematodes point to a perturbation of a complement receptor on human immune cells [37]–[40]. Here, we demonstrate that plant-parasitic nematodes most likely deliver venom allergen-like proteins into the apoplast of host cells to suppress basal immunity mediated by surface-localized immune receptors.

Several lines of evidence in our data suggest that plant-parasitic nematodes may use venom allergen-like proteins to modulate immune responses activated by tissue damage caused by migratory nematodes inside host plants. First, the expression of *Gr-VAP1* in *G. rostochiensis* coincides with host invasion by infective juveniles and intracellular migration by adult males inside plants (Fig. 2). Both host invasion and migration by parasitic nematodes involves the enzymatic breakdown of plant cell walls, resulting in extensive damage to host tissue [56]. More importantly, Gr-VAP1 is secreted at the same time and from the same pharyngeal glands in *G. rostochiensis* as an elaborate set of plant cell wall-degrading enzymes [14]. The transient knockdown of these plant cell wall-degrading enzymes in infective juveniles of *G. rostochiensis* inhibits the onset of parasitism [56]. Similarly, the transient knockdown of *Gr-VAP1* expression during host invasion significantly reduced the number of infective juveniles inside susceptible tomato plants (Fig. 1), showing that this venom allergen-like protein is also a critical factor during the onset of parasitism.

Although we have not formally shown that plant-parasitic nematodes deliver VAPs into the apoplast of host cells in planta, our previous work demonstrates that this is nonetheless most likely the case [14]. More specifically, we have shown that Gr-VAP1 associates with and perturbs apoplastic Rcr3^pim^ in tomato, and that this perturbation specifically activates nematode resistance and programmed cell death mediated by the extracellular receptor-like protein Cf-2. Furthermore, tomato plants that harbor apoplastic Rcr3^pim^, but not Cf-2, are almost twice as susceptible to nematode infections than tomato plants with allelic variants of apoplastic Rcr3 to which Gr-VAP1 is unable to bind or tomato plants that have no functional Rcr3 at all. Altogether, we conclude that venom allergen-like proteins may specifically function as apoplastic suppressors of immune responses triggered by plant cell wall fragments released by the enzymatic breakdown of plant cell walls during nematode migration inside host plants [57], [58].

Little work has been done on the importance of surface-localized pattern recognition receptors mediating damage-triggered immunity in nematode-plant interactions. Transcriptome analyses of nematode-infected roots suggest that plant cell wall-associated legume-like lectin receptor kinases might be involved in basal immunity to *H. schachtii* in Arabidopsis [59], but further research is needed to corroborate this. Interestingly, both Hs-VAP1 and Hs-VAP2 significantly down-regulate the expression of five proline-rich extensin-like receptor kinases (i.e. At4G34440, *AtPERK5*; At3G18810, *AtPERK6*; At1G49270, *AtPERK7*; At1G10620, *AtPERK11*; S1 Table and S2 Table)). These PERKs belong to a family of fifteen predicted transmembrane receptor-like kinases in Arabidopsis. The extracellular domain in PERKs shares similarity with plant cell wall-associated extensin proteins [60], but the biological function of most members of the *AtPERK* family is unknown. However, the expression of *BnPERK1* from *Brassica napus* is rapidly induced following wounding, because of which it is thought to mediate early events in defense responses to cell wall damage by invading plant pathogens [61].

The modulation of basal immunity by venom allergen-like proteins in plants most likely involves at least two different classes of extracellular proteases. The first class of extracellular proteases regulating basal immunity to nematode infections in plants concerns the papain-like cysteine proteases. The inhibition of the extracellular papain-like cysteine protease Rcr3^pim^ from *S. pimpinellifolium* by Gr-VAP1 results in enhanced susceptibility of tomato plants to *G. rostochiensis* [14]. Here, we showed that Gr-VAP1 also perturbs the extracellular papain-like cysteine protease C14^tub^ from potato (*S. tuberosum*; Fig. 3B), while ectopic Gr-VAP1 significantly increased the susceptibility of potato plants to *G. rostochiensis* (Fig. 3A). Remarkably, ectopic Gr-VAP1 also suppressed basal immunity of Arabidopsis, even though this plant species is not a host of *G. rostochiensis*. However, a similar phenomenon has been observed with the apoplastic effector Avr2 of the *C. fulvum*, which acts as an inhibitor of Rcr3^pim^ and several other extracellular papain-like cysteine proteases in tomato [15], [16], [42], [62]. Although *A. thaliana* is not a host of *C. fulvum* either, ectopic Avr2 nonetheless interacts with multiple papain-like cysteine proteases required for basal defense in Arabidopsis [42]. The inhibition of apoplastic papain-like cysteine proteases by ectopic Avr2 also suppresses immunity of Arabidopsis to *H. schachtii* (Fig. 5A). Similarly, the lack of three papain-like cysteine proteases most related to Rcr3^pim^ in Arabidopsis mutants (i.e. *pap1*, *pap4*, and *pap5*) suppresses immunity to *H. schachtii* (Fig. 5B) Altogether, these findings position the inhibition of extracellular papain-like cysteine proteases by apoplastic effectors as an important regulatory process in plant innate immunity to cyst nematodes.

The second class of extracellular proteases most likely involved in the suppression of basal immunity by venom allergen-like proteins concerns subtilisin-like serine proteases. The transcript most down-regulated by ectopic Hs-VAP1 in Arabidopsis encodes the plant cell wall-associated subtilisin-like serine protease AtSBT3.14 (S1 Table; AT4G21630; [49], [63]). *AtSBT3.14* is also down-regulated by ectopic Hs-VAP2, albeit to a lesser extent. The Arabidopsis SBT family comprises 56 members, most of which are still uncharacterized. *AtSBT3.14* is specifically expressed in roots, siliques, and dry seed of *A. thaliana*, but its function is not known [63]. AtSBT3.14 belongs to the same subfamily as AtSBT3.3, which functions as an extracellular molecular switch in the priming of defense responses in Arabidopsis [50]. T-DNA insertion knockout mutations in the *AtSBT3.3* gene compromise innate immunity of Arabidopsis. The loss of immunity of Arabidopsis mutants lacking a functional *AtSBT3.14* gene to *H. schachtii* suggests that this plant cell wall-associated subtilase may also act as an extracellular regulator of basal innate immunity (Fig. 6).

Most of the genes differentially regulated by the overexpression of venom allergen-like proteins in Arabidopsis are typically associated with innate immunity and plant cell wall-associated processes. A notable exception to this is *NPQ4,* which was the most up-regulated gene in both *Hs-VAP1* and *Hs-VAP2* overexpressing plants. The PsbS protein encoded by *NPQ4* is involved in non-photochemical fluorescence quenching in the thylakoid membranes of chloroplasts [51], [64]. PsbS functions as photo protectant by mediating the thermal dissipation of excess excitation energy of singlet chlorophyll. Saturation of the electron transport chain in the photosystem II by excess light can lead to the accumulation of excited chlorophyll, which when insufficiently quenched by PsbS transfers its energy to oxygen to form highly reactive singlet oxygen [65]. The non-photochemical quenching capacity in chloroplasts thus regulates the generation of singlet oxygen [54]. Singlet oxygen can be the cause of oxidative damage, but on the other hand it is also involved in the peroxidation of lipids into oxylipin hormones (e.g. jasmonic acid; [54]) and in the onset of programmed cell death [55], [66]. It is for this duality that PsbS is thought to play a key role in the trade-off between the ability to protect against abiotic photo-oxidative stress and the ability to mount effective redox-dependent immune responses to biotic invaders [54].

Our data shows that ectopic Hs-VAP1 and Hs-VAP2 suppress innate immune responses in Arabidopsis, at least partly, through their regulation of PsbS. As PsbS is a rate-limiting factor in non-photochemical quenching of excited singlet chlorophyll [67], the more than 30-fold increase in the expression of *NPQ4* by ectopic venom allergen-like proteins in the transgenic Arabidopsis most likely reduces the formation of singlet oxygen under biotic stress [54]. As a consequence, the constitutive augmentation of the non-photochemical quenching capacity by elevated levels of PsbS will probably also affect the production of oxylipin hormones (i.e. jasmonic acid, and its precursors) and the signaling of programmed cell death in response to biotic stress [54]. By contrast, PsbS-deficient *npq4-1* mutant Arabidopsis plants show an enhanced production of jasmonic acid in response to herbivory by feeding insects [68]. We used the same Arabidopsis mutant line to demonstrate that PsbS-deficient plants are immune to infections by *H. schachtii* (Fig. 6). This finding shows that a functional PsbS protein is required for virulence of *H. schachtii* in Arabidopsis, possibly for down-regulating oxylipin hormone signaling or other singlet oxygen-dependent immune responses.

While a PsbS-centered model may offer a plausible explanation for the suppression of immune responses by ectopic venom allergen-like proteins in leafs, the biological relevance of PsbS as regulator immunity to parasitic nematodes in roots is more puzzling. PsbS is localized in chloroplasts, which mainly occur in aerial plant parts that are exposed to light but not in roots. However, the Arabidopsis plants used in nematode infection assays are routinely cultured in vitro on translucent media in a light/dark cycle to monitor the infection over time. It has been shown before that permanent feeding structures formed by *H. schachtii* under these circumstances harbor chloroplasts [69]. Importantly, it has also been shown that *NPQ4* is strongly up-regulated in permanent feeding structures of *H. schachtii* as compared to other root cells in the elongation zone of Arabidopsis plants kept in a light/dark cycle [70]. One could argue that the occurrence of chloroplasts and the expression of *NPQ4* in nematode-induced feeding structures in roots are artifacts caused by the unnatural exposure of the roots to light. However, others have shown that permanent feeding structures of *H. schachtii* in Arabidopsis roots that are kept in the dark also harbor plastids with similar fluorescence spectra as chloroplasts [70]. More research is therefore needed to further investigate the nature and functions of PsbS in plastids of nematode-infected roots. Taken together, we conclude that non-photochemical quenching capacity is at least partly responsible for regulating innate immunity of roots to infection by *H. schachtii* under our experimental conditions.

In conclusion, plants monitor the integrity of their cell walls with specific surface-localized pattern recognition receptors [71], [72]. The recognition of fragments of plant cell walls can elicit strong basal defense responses that counteract further invasion by microbial invaders [73], [74]. As plant-parasitic nematodes cause significant damage to plant cell walls during their migration inside host plants, they could evidently benefit from effectors that suppress immunity triggered by fragments from damaged plant cell walls. Our data allows for a model in which apoplastic venom allergen-like proteins of plant-parasitic nematodes suppress host defenses activated by the detection of fragments of plant cell walls released by migrating nematodes. This model could also explain why ectopic VAPs particularly affect the susceptibility of Arabidopsis to diverse unrelated lesion-forming plant pathogens, the symptoms of which also involve significant plant cell wall modifications.

So far, we have identified three components of the molecular mechanisms that are most likely underlying the suppression of plant innate immunity by apoplastic venom allergen-like proteins (i.e. extracellular papain-like cysteine proteases, cell wall-associated subtilisin-like serine protease, and the chlorophyll-associated Photosystem II subunit S protein). As our mutant analyses showed, each of these components separately has a major impact on immunity to cyst nematodes in Arabidopsis. An important question that needs further research is if all three components are part of a single signaling pathway that spans different subcellular compartments. This might not be the case, because promiscuous effectors like Gr-VAP and Avr2 can interact with multiple apoplastic papain-like cysteine proteases, each of which may feed into different signaling pathways.

## Materials and Methods

### Knockdown of *Gr-VAP1* expression by RNA interference

*Gr-VAP1* expression in preparasitic second stage juveniles (ppJ2s) of *G. rostochiensis* was knocked-down by soaking nematodes in double-stranded (ds) RNA matching 820 base pairs of the *Gr-VAP1* coding sequence as described by Chen et al [75] and Rehman et al [56]. Briefly, a cDNA fragment was PCR-amplified with the primers Gr-VAP1-RNAiFW and Gr-VAP1-RNAiR (S7 Table) using Gr-VAP1 cDNA as template. The amplified cDNA fragment of Gr-VAP1 was subsequently used as template for generating dsRNA in vitro using the Megascript RNAi kit (Ambion, Cambridgeshire, UK). Double-stranded RNA matching the sequence of the *Nautilus* gene from *Drosophila melanogaster* (Genbank accession number M68897) was used as control treatment. RNA interference was induced in nematodes by soaking approximately 15,000 freshly hatched ppJ2s of *G. rostochiensis* Ro1 Mierenbos in a 1 mg/ml dsRNA solution, including 50 mM octopamine, 3 mM spermidine, and 0.05% gelatin. The treatments were done *in duplo* so that 15,000 juveniles could be processed further for infectivity assay on tomato seedlings and 15,000 juveniles could be used for semi-quantitative reverse transcription (RT)-PCR.

To test the effect of RNA interference on infectivity of *G. rostochiensis*, we inoculated plates with five two-week old tomato seedlings (cultivar Moneymaker) on Gamborg B5 medium with 400 dsRNA-treated ppJ2s [56]. For each treatment a total number of 10 plates was inoculated with dsRNA-treated ppJ2s. The plants were grown at 24°C and light/dark cycles of 16 h/8 h. Seven days post inoculation, the roots were stained with acid fuchsin, destained using acidified glycerol, and the number of nematode per root was determined using a dissection microscope. The means of numbers of nematodes per plant were tested for significant differences in a one-way ANOVA.

To analyze *Gr-VAP1* expression after dsRNA treatment, total RNA was extracted from dsRNA-treated ppJ2s using the RNeasy Mini kit (Qiagen, Hilden, Germany). RT-PCR was done following the protocol of the SuperScript™ III One-Step RT-PCR System (Invitrogen) using the primer Gr-VAP1-sRTFw and Gr-VAP1-sRTRv (S7 Table), which were designed outside the region targeted by the dsRNA. The expression of the 60S acidic ribosomal protein-encoding gene (Genbank accession number BM354715.1) was analyzed with primers 60S-RTFw and 60S-RTRv (S7 Table) as a reference for constitutive expression. Aliquots of the PCR solutions were visualized on ethidium bromide stained 1% agarose gel after 28 cycles.

### Fluorescent protease activity profiling

Fluorescent activity based protease profiling was used to test whether Gr-VAP1 perturbs the active site of cysteine proteases. The cysteine proteases *C14^tub^* of *S. tuberosum* and *C14^lyc^* of *S. lycopersicum* were transiently overexpressed in apoplastic fluids of *N. benthamiana* leaves following agroinfiltration [62]. Twenty-five to fifty microliters of apoplastic fluid was incubated with either 100 nM of *P. pastoris* produced Avr2, 100 nM cystatin from chicken egg-white (Sigma-Aldrich), or 300 nM of Gr-VAP1 isolated from apoplastic fluids of agroinfiltrated *N. benthamiana* leaves (see below) in 50 mM sodium acetate (pH 5.5) and 100 µM DTT. To label the available active sites in these cysteine proteases, the proteins were subsequently incubated for 5 h with 1 µM of fluorescent DCG-04-TMR [76]. Fluorescent labeled proteins were separated in 12% Bis-Tris gels (Invitrogen), which were subsequently analyzed using a fluorescent imager scanner (Molecular Imager FX, Bio-Rad, Hercules, CA, USA). Labelling densities were quantified in triplicates using the computer software Quantity One 4.6.9 (Bio-Rad Laboratories).

### Temporal expression of *Gr-VAP1* in *G. rostochiensis*

To study the expression of *Gr-VAP1* at different time points before and after inoculation, we used semi-quantitative RT-PCR as described above. Messenger RNA extraction and cDNA synthesis was conducted on parasitic second, third, and fourth stage juveniles and the adult males and females isolated from roots of susceptible potato (cultivar Bintje) at 13, 19, 23, 27, and 34 days post inoculation respectively. *Gr-VAP1* expression in these developmental stages was examined by targeting a gene specific fragment of 146 base pairs of Gr-VAP1 with primers Gr-VAP1-RTFw and Gr-VAP1-RTRv (S7 Table). The constitutively expressed cAMP-dependent protein kinase (*Gr-cAMP*; GenBank accession number BM343563) was PCR amplified with the primers cAMP-RTFw and cAMP-RTRv (S7 Table) as a reference. We included reactions without reverse transcriptase to test for contaminating genomic DNA of the nematodes, while non-infected potato roots were included to check for non-specific amplification of host-derived cDNA.

### Generating transgenic potato plants

Transgenic potato plants (Line V; genotype 6487-9) overexpressing Gr-VAP1 in the apoplast were generated as described by Postma et al [30]. Briefly, potato stem pieces were incubated for 10 minutes with a suspension of *Agrobacterium tumefaciens* strain AGL1 carrying the *Gr-VAP1* cDNA sequence, including its native signal peptide for secretion, in pMDC32 [14], [77]. Transformant callus was selected on ZCVK medium (MS20 medium, 8 g/l plant agar, 1 mg/l zeatin, 100 mg/l kanamycin, 200 mg/l cefotaxim, 200 mg/l vancomycin; pH 5.8). The introgression of *Gr-VAP1* insert was checked by PCR on genomic DNA extracted from plant leaves using the DNeasy Plant Mini Kit (Qiagen). The expression of *Gr-VAP1* was checked by quantitative PCR (qPCR) using the primers qGrVAP1-Fw and qGrVAP1-Rv (S7 Table) on RNA extracted from leaves using the RNeasy Plant Mini Kit (Qiagen). The constitutively expressed actin was amplified with the primers StActinF and StActinR (S7 Table) as a reference. qPCR was performed using Absolute QPCR SYBR Green Mix (Thermo Fisher Scientific) with amplification of 15 min at 95°C, followed by 35 cycles of 30 s at 95°C, 30 s at 63°C and 30 s at 72°C.

### Infection assays on potato plants

Dried cysts of *G. rostochiensis* pathotype Ro1-Mierenbos were soaked in potato root diffusate on a 100-µm sieve to collect ppJ2s [78]. Remnants of roots and other debris were removed from suspensions of freshly hatched ppJ2s using centrifugation in sucrose gradient. Prior to inoculation potato plants the ppJ2s were surface sterilized, and resuspended in sterile 0.7% (w/v) solution of Gelrite (Duchefa) as previously described [30]. Approximately, 200 ppJ2s were inoculated onto 3-week-old *in vitro*-grown plants potato plant. Adult females per plant were counted 6 to 8 weeks after inoculation. Two independently transformed potato lines were used in these experiments. The infection assays were repeated at least 3 times.

### Identification and cloning of venom allergen-like protein from *H. schachtii*

To identify and clone venom allergen-like proteins from the beet cyst nematode *H. schachtii*, we first queried the expressed sequence tag database at Genbank using the sequence of Gr-VAP1 as query. Four cDNA library clones, from which matching expressed sequence tags derived, were acquired from “The Washington University Nematode EST Project”[79]. Re-sequencing of library insert in these clones, with the primers M13Fw and M13Rv (S7 Table), resulted in the identification of two full-length cDNA sequences encoding novel venom allergen-like proteins named Hs-VAP1 and Hs-VAP2. The cDNA sequences encoding the complete open reading frames of Hs-VAP1 and Hs-VAP2, including native signal peptides for secretion, were PCR- amplified with gene specific primers Hs-VAP1-GWFw, HsVAP1-GwRv, HsVAP2-GWFw, and HsVAP2-GwRv (S7 Table) and cloned into the entry vector pENT/D-TOPO (Invitrogen). The inserts in these entry vectors were subcloned into the binary plasmids pMDC32 for stable plant transformation [77] or pGWB411 [80] for transient expression, using Gateway technology (Invitrogen).

### Generation of transgenic Arabidopsis

To generate transgenic Arabidopsis lines constitutively overexpressing venom allergen-like proteins in apoplast, we transformed *A. thaliana* Columbia 0 with *A. tumefaciens* strain GV3101 carrying constructs of Gr-VAP1, Hs-VAP1, and Hs-VAP2 in pMDC32, and pMDC32 without insert, using the floral dip method [81]. Primary transformants were selected on agar with 50 µg/ml kanamycin after which the plants were transferred to soil to produce seeds. Several independent homozygous single insertion lines were selected, and T3 and T4 generations were used for infection assays (see below). The introgression of *Hs-VAP1* and *Hs-VAP2* was checked by PCR on genomic DNA extracted from seedlings using the DNeasy Plant Mini Kit (Qiagen). The expression of the transgenes was checked by qPCR using the primers qHsVAP1-F, qHsVAP1-R, qHsVAP2-F, and qHsVAP2-R (S7 Table) on RNA extracted from seedlings using the RNeasy Plant Mini Kit (Qiagen). The clathrin adaptor complex medium subunit family protein (At5g46630) was amplified with the primers AtClathrinF and AtClathrinR (S7 Table) as a reference. qPCR was performed using Absolute QPCR SYBR Green Mix (Thermo Fisher Scientific) with amplification of 15 min at 95°C, followed by 35 cycles of 30 s at 95°C, 30 s at 60°C and 30 s at 72°C.

Seeds of the homozygous transgenic T-DNA insertion mutants of the cysteine proteases *PAP1* (At2g34080), *PAP4* (At2g27420), *PAP5* (At3g49340), the serine protease *SBT3.13* (At4g21630), and the *chlorophyll-associated Photosystem II subunit S* (At1g44575) were obtained from the SALK homozygote T-DNA collection. The mutant plants were propagated under standard greenhouse conditions of a 16-h/8-h light/dark regime and 60% relative humidity.

### Infection assays on Arabidopsis plants

Seeds from transgenic *Arabidopsis* and wild-type *A. thaliana* Col-0 were vapor sterilized and planted in 12-well cell culture plates (Greiner bio-one) containing modified Knop's medium [82]. Plants were grown at 24°C under 16-h-light/8-h-dark conditions. Two-week-old seedlings were inoculated with ∼250 surface-sterilized ppJ2s of *H. schachtii* [83]. Two and four weeks after inoculation, the number of female J4s of *H. schachtii* was counted by visual inspection. The statistical significance of the pairwise differences between plant genotypes and the empty pMDC32 vector control and the wild type Arabidopsis was assessed with a one-way ANOVA.

The susceptibility of the *Arabidopsis* plants to infections by *B. cinerea*, *P. cucumerina*, *A. brassicicola*, and *P. brassicae* was determined on 4-week-old soil-grown plants [42], [84], [85]. Briefly, for *B. cinerea*, *P. cucumerina*, *A. brassicicola*, plants were drop inoculated by placing two 4-µl drops of conidial suspension (5×10^5^ conidia/ml) on each leaf. Plants were incubated at 20°C, 100% relative humidity, and a 16-h/8-h light/dark regime. Arabidopsis was inoculated with *P. brassicae* by placing 5-mm-diameter mycelial plugs of a 2-week-old *P. brassicae* agar plate culture onto leaves. Subsequently, the plants were incubated at 16°C, 100% relative humidity, and a 16-h/8-h light/dark regime. After two days the mycelial plugs were removed from the leaves. Disease progression for these pathogens was scored at regular intervals, and representative pictures were taken at 4 days after inoculation. The statistical significance of the pairwise differences between plant genotypes and the empty pMDC32 vector control was assessed with a one-way ANOVA.

For inoculation of *Arabidopsis* with *V. dahliae*, 2-week-old soil-grown plants were uprooted and inoculated by dipping the roots for 2 min in a conidial suspension (10^6^ conidia/ml). After replanting in soil, plants were incubated at standard greenhouse conditions of a 16-h/8-h light/dark regime and 60% relative humidity. Disease progression was monitored until 25 days after inoculation. The statistical significance of the pairwise differences between plant genotypes and the empty pMDC32 vector control was assessed with a one-way ANOVA. All infection assays were performed at least 2 times.

The susceptibility of the *Arabidopsis* plants to infections by *P. syringae* pv. *tomato* DC3000 was determined on 2-week-old Arabidopsis seedlings as previously described [86]. Briefly, three 2-µl drops of a cell suspension of *Pst* at 10^9^ CFU/ml, in 10 mM MgSO_4_ supplemented with 0.01% (v/v) Silwet L77, was inoculated on the two most expanded and in the center of the leaf rosette. Inoculated plants were subsequently incubated at 21°C, 100% relative humidity, and a 16-h/8-h light/dark regime. Disease severity was scored 3 days after challenge inoculation. Colonization levels of the bacteria were determined with the method described by Pieterse *et al*. [87]. The statistical significance of the pairwise differences between plant genotypes and the empty pMDC32 vector control was assessed with a one-way ANOVA.

### Growth inhibition assays

Arabidopsis growth inhibition assays were performed as described elsewhere [88]. Briefly, seedlings were grown for 5 days on MS agar plates, supplemented with 1% w/v sucrose and 0.8% agar. Subsequently, seedlings were transferred to liquid MS medium supplied with 10 µM of the flg22 (QRLSTGSRINSAKDDAAGLQIA) synthetic peptide. One seedling was placed on 400 µl of medium in wells of 24-well-plates. The effect of treatment with the flg22 peptide on the growth of transgenic and wild type Arabidopsis (Col-0) seedlings was analyzed after 7 days by measuring root length. Statistical significance of the difference between plant genotypes was assessed with a one-way ANOVA.

### RNA-seq on Arabidopsis plants

Two-weeks-old transgenic *Arabidopsis* plants, grown under the same conditions as for the infection with *H. schachtii*, were collected, flash-frozen in liquid nitrogen and total RNA was extracted with the Maxwell® 16 LEV simplyRNA purification kit (Promega). cDNA synthesis, library preparation (200-bp inserts), and Illumina sequencing (90-bp paired-end reads) was performed at BGI (Hong-Kong). Reads were mapped to the Arabidopsis genome (tair10) using TopHat and transformed into a count per gene per sample by using the BEDTools suite (function coverageBed). The edgeR [89] method was used to analyze differentially expressed genes (DEGs) between groups. DEGs were mapped to Gene Ontology (GO) terms in the database (http://www.geneontology.org/), and gene numbers were calculated for every term using an ultra-geometric test to find significantly enriched GO terms in DEGs. Calculated p-value went through a Bonferroni Correction, taking corrected p-value ≤0.05 as a threshold. KEGG pathway enrichment analysis was used to identify significantly enriched metabolic pathways or signal transduction pathways in DEGs comparing with the whole genome background. Subcellular localization was determined for all DEGs using the SUBcellular localization database for Arabidopsis proteins [90].

### Suppression of defense-related programmed cell death in *Nicotiana benthamiana*

The suppression of programmed cell death in leaves of *N. benthamiana* was assessed by using Gr-VAP1, Hs-VAP1, Hs-VAP2, and Mi-VAP1 (including their native signal peptide for secretion) subcloned into pGWB411 [80]. The Mi-VAP1 construct was synthetized at GeneArt (Life Technologies) based on the sequence in Genbank (accession AAD01511.1). All constructs were transferred to *Agrobacterium tumefaciens* GV3101, and used for agroinfiltration in leaves of the *N. benthamiana*. Empty pGWB411 and plasmids carrying GFP in pGWB411 [80] were used as controls to assess the non-specific suppression of programmed cell death by agroinfiltration. The transient co-expression by agroinfiltration of several pairs of resistance genes and cognate elicitors was used to induce programmed cell death in leaves of *N. benthamiana* (S6 Table; [30]). *A. tumefaciens* harboring individual binary vectors was grown at 28°C in liquid yeast extract peptone medium with appropriate antibiotics for 16 h. The bacteria were spun down and resuspended in infiltration medium to an optical density at 600 nm (OD600) of 0.1 [30]. Agroinfiltration was done the abaxial side of the leaves of *N. benthamiana* using a 1 ml syringe. Co-infiltration of different constructs was performed by mixing equal volumes of the bacterial suspensions to a final optical density of 0.3. Agroinfiltrated leaves were monitored for up to 7 d for cell death symptoms.

To assess whether the VAPs harboring their native signal peptide for secretion (in pGWB411) were indeed secreted to the apoplast of agroinfiltrated leaves of *N. benthamiana*, we isolated apoplastic fluids by vacuum-infiltrating ice-cold extraction buffer (50 mM phosphate-buffered saline pH = 7.4, 100 mM NaCl, and 0.1% v/v Tween-20) for 10 min. Infiltrated leaves were surface dried and placed in a 10-ml syringe hanging in a 50 ml tube and centrifuged at 2000 *g* for 10 min at 4°C. Apoplastic fluids were subsequently separated under reducing conditions by SDS-PAGE on a 12% Bis-Tris gel and transferred to an Invitrolon™ PVDF membrane (Life Technologies). For visualization of VAPs on western blots, we used a mouse monoclonal ANTI-FLAG^®^ M2-Peroxidase (HRP) antibody to detect the FLAG tag at the carboxyl terminus of the recombinant proteins. Pictures were taken using the G:BOX Chemi System device (SynGene).

## Supporting Information

S1 Figure **Apoplastic Gr-VAP1 perturbs the active site of the extracellular defense-related papain-like cysteine protease C14^tub^ of potato (** ***Solanum tuberosum*** **).** Labeling densities of the fluorescent activity-based probe DCG-04 to the active site of (A) C14^tub^ and (B) C14^lyc^ of tomato (*S. lycopersicum*) following treatment with Gr-VAP1 isolated from apoplastic fluids of agroinfiltrated leaves. Treatments with the Avr2, egg white cystatin, and apoplastic fluids from agroinfiltrations with the empty binary expression vector (EV), and with buffer alone (Buffer) were included as controls. Labeling densities were quantified in triplicates and statistical significance of differences was determined with an ANOVA. Different letters indicate significant differences when using of *P*-value <0.05 as threshold.
(TIF)

S2 Figure **Protein sequence variation in venom allergen-like proteins from cyst nematodes and root-knot nematodes.** (A) Protein sequence alignment of venom allergen-like proteins from the potato cyst nematode *Globodera rostochiensis* (Gr-VAP1), the beet cyst nematode *Heterodera schachtii* (Hs-VAP1 and Hs-VAP2), and the root-knot nematode *Meloidogyne incognita* (Mi-VAP1). Colors indicate identity (black background) or similarity among the sequences (gray background). (B) Protein similarity matrix of venom allergen-like proteins. Numbers represent the percentage of amino acid residues that are similar (bottom left corner) and identical for any pair of proteins.
(TIF)

S3 Figure **Ectopic venom allergen-like proteins enhance susceptibility to** ***Pseudomonas syringae*** **pv.** ***tomato*** **(** ***Pst*** **) in Arabidopsis.** Heterologous expression of the venom allergen-like proteins Hs-VAP1 and Hs-VAP2 from *Heterodera schachtii* in the apoplast of transgenic Arabidopsis lines enhances their susceptibility to *Pst* DC3000. Two independent transgenic lines per construct (-A and -B) were compared with corresponding transgenic line harboring the T-DNA of the empty vector (EV) and wild type *A. thaliana* (Col-0). (A) Population densities were determined 4 days after inoculation with *Pst*. Bars represent colony forming units (cfu/mg of tissue) for three independent replicates of 8 plants each. (B) Disease incidence was evaluated 4 days after inoculation. Bars represent the mean percentage of leaves, which had developed chlorotic symptoms of 24 plants. Statistical significance of differences was determined with an ANOVA. Different letters indicate statistical significance when using *P*-value <0.05 as threshold. (C) Pictures show typical symptoms on Arabidopsis plants inoculated either with *Pst*, or mock inoculated.
(TIF)

S4 Figure **Ectopic venom allergen-like proteins enhance development of disease symptoms of fungal and oomycete pathogens in Arabidopsis.** Bars represent mean percentage infected leaf area of transgenic Arabidopsis line overexpressing Hs-VAP1 and Hs-VAP2 3 days after inoculation with (A) *Botrytis cinerea*, (B) *Plectosphaerella cucumerina*, and (C and D) two isolates of *Phytophthora brassicae* (CBS686.95 and HH). Statistical significance of differences with transgenic plants harboring the T-DNA of the corresponding empty expression vector (EV) and wild type Arabidopsis (Col-0) was determined with an ANOVA. Different letters indicate significant differences when using of *P*-value <0.05 as threshold.
(TIF)

S5 Figure **Ectopic venom allergen-like proteins abrogate the inhibition of seedling growth by flg22 in Arabidopsis.** Photograph of typical root length of transgenic Arabidopsis lines overexpressing Hs-VAP1 and Hs-VAP2 after 10 days of growth in the presence of 10 µM flg22. Transgenic plants harboring the T-DNA of the corresponding empty expression vector (EV) and wild type Arabidopsis (Col-0) were used to show the normal inhibition of root growth in the presence of flg22.
(TIF)

S6 Figure **Ectopic venom allergen-like proteins regulate immunity related pathways in Arabidopsis.** Global gene expression analysis as determined by RNA-seq in 2 weeks old Arabidopsis plants overexpressing Hs-VAP1 and Hs-VAP2 in the apoplast. Venn's diagrams depict the total number of significantly up- (A) and down-regulated (B) genes relative to transgenic Arabidopsis plants harboring T-DNA of the corresponding empty expression vector (EV) when using a false discovery rate of 0.05 as cutoff. (C) Pie charts depict percentage of products of genes significantly down-regulated by ectopic Hs-VAP1 and Hs-VAP2 in Arabidopsis, according to their predicted subcellular localization in the SUBA database.
(TIF)

S7 Figure **VAPs harboring their native signal peptide for secretion are secreted to the apoplast of agroinfiltrated leaves of** ***Nicotiana benthamiana*** **.** *Heterodera schachtii* VAPs (Hs-VAP1 and –VAP2), *Globodera rostochiensis* VAP1 (Gr-VAP1), and *Meloidogyne incognita* VAP1 (Mi-VAP1) were transiently expressed in *N. benthamiana* plants as recombinant carboxyl terminus FLAG tagged proteins together with empty vector (EV) controls. VAPs were detected in apoplastic fluids isolated from agroinfiltrated leaf segments at 5 days post infiltration on western blots using FLAG specific antibody.
(TIF)

S1 Table **Most down-regulated transcripts by ectopic venom allergen-like proteins in Arabidopsis.** Differentially expressed genes in transgenic *Arabidopsis thaliana* overexpressing Hs-VAP1 relative to the corresponding transgenic empty vector control plants.
(XLSX)

S2 Table **Most down-regulated transcripts by ectopic venom allergen-like proteins in Arabidopsis.** Differentially expressed genes in transgenic *Arabidopsis thaliana* overexpressing Hs-VAP2 relative to the corresponding transgenic empty vector control plants.
(XLSX)

S3 Table **Most up-regulated transcripts by ectopic venom allergen-like proteins in Arabidopsis.** Differentially expressed genes in transgenic *Arabidopsis thaliana* overexpressing Hs-VAP1 relative to the corresponding transgenic empty vector control plants.
(XLSX)

S4 Table **Most up-regulated transcripts by ectopic venom allergen-like proteins in Arabidopsis.** Differentially expressed genes in transgenic *Arabidopsis thaliana* overexpressing Hs-VAP2 relative to the corresponding transgenic empty vector control plants.
(XLSX)

S5 Table **KEGG pathway enrichment analysis: Statistically enriched pathways among differentially expressed genes in *Arabidopsis thaliana* overexpressing *Hs-VAP1* and *Hs-VAP2* relative to the corresponding transgenic empty vector plants.**
(DOCX)

S6 Table **Plant immune receptors and cognate pathogen elicitors used to induce programmed cell death in leaves of *Nicotiana benthamiana*.**
(DOCX)

S7 Table **Oligonucleotide primers used in this study.**
(DOCX)

## References

[1] Jones JT, Haegeman A, Danchin EGJ, Gaur HS, Helder J, et al. (2013) Top 10 plant-parasitic nematodes in molecular plant pathology. Mol Plant Pathol: 946–961. 10.1111/mpp.12057 PMC6638764 23809086

[2] McCarter JP (2009) Molecular approaches toward resistance to plant-parasitic nematodes. In: Berg RH, Taylor CG, editors. Cell biology of plant nematode parasitism.Berlin Heidelberg: Springer Verlag. pp.239–267.

[3] Goverse A, Smant G (2014) The Activation and Suppression of Plant Innate Immunity by Parasitic Nematodes. Annu Rev Phytopathol 52: in press. 10.1146/annurev-phyto-102313-050118 24906126

[4] Williamson VM, Kumar A (2006) Nematode resistance in plants: the battle underground. Trends Genet 22: 396–403. 16723170 10.1016/j.tig.2006.05.003

[5] Gish LA, Clark SE (2011) The RLK/Pelle family of kinases. Plant J 66: 117–127. 21443627 10.1111/j.1365-313X.2011.04518.x PMC4657737

[6] Boller T, Felix G (2009) A renaissance of elicitors: Perception of microbe-associated molecular patterns and danger signals by pattern-recognition receptors. Annu Rev Plant Biol 60: 379–407. 19400727 10.1146/annurev.arplant.57.032905.105346

[7] Schwessinger B, Zipfel C (2008) News from the frontline: recent insights into PAMP-triggered immunity in plants. Curr Opin Plant Biol 11: 389–395. 18602859 10.1016/j.pbi.2008.06.001

[8] Chisholm ST, Coaker G, Day B, Staskawicz BJ (2006) Host-microbe interactions: Shaping the evolution of the plant immune response. Cell 124: 803–814. 16497589 10.1016/j.cell.2006.02.008

[9] Kruijt M, De Kock MJD, De Wit PJGM (2005) Receptor-like proteins involved in plant disease resistance - Review. Mol Plant Pathol 6: 85–97. 20565641 10.1111/j.1364-3703.2004.00264.x

[10] Dixon MS, Jones DA, Keddie JS, Thomas CM, Harrison K, et al (1996) The Tomato Cf-2 Disease Resistance Locus Comprises Two Functional Genes Encoding Leucine-Rich Repeat Proteins. Cell 84: 451–459. 8608599 10.1016/s0092-8674(00)81290-8

[11] Wang G, Fiers M, Ellendorff U, Wang Z, de Wit PJGM, et al (2010) The diverse roles of extracellular leucine-rich repeat-containing receptor-like proteins in plants. Crit Rev Plant Sci 29: 285–299.

[12] Liebrand TWH, van den Burg HA, Joosten MHAJ (2014) Two for all: receptor-associated kinases SOBIR1 and BAK1. Trends Plant Sci 19: 123–132. 24238702 10.1016/j.tplants.2013.10.003

[13] Monaghan J, Zipfel C (2012) Plant pattern recognition receptor complexes at the plasma membrane. Curr Opin Plant Biol 15: 349–357. 22705024 10.1016/j.pbi.2012.05.006

[14] Lozano-Torres JL, Wilbers RHP, Gawronski P, Boshoven JC, Finkers-Tomczak A, et al (2012) Dual disease resistance mediated by the immune receptor Cf-2 in tomato requires a common virulence target of a fungus and a nematode. Proc Natl Acad Sci U S A 109: 10119–10124. 22675118 10.1073/pnas.1202867109 PMC3382537

[15] Krüger J, Thomas CM, Golstein C, Dixon MS, Smoker M, et al (2002) A tomato cysteine protease required for Cf-2-dependent disease resistance and suppression of autonecrosis. Science 296: 744–747. 11976458 10.1126/science.1069288

[16] Rooney HCE, Van't Klooster JW, Van Der Hoorn RAL, Joosten MHAJ, Jones JDG, et al (2005) *Cladosporium* Avr2 inhibits tomato Rcr3 protease required for Cf-2-dependent disease resistance. Science 308: 1783–1786. 15845874 10.1126/science.1111404

[17] Song J, Win J, Tian M, Schornack S, Kaschani F, et al (2009) Apoplastic effectors secreted by two unrelated eukaryotic plant pathogens target the tomato defense protease Rcr3. Proc Natl Acad Sci U S A 106: 1654–1659. 19171904 10.1073/pnas.0809201106 PMC2635833

[18] Cantacessi C, Campbell BE, Visser A, Geldhof P, Nolan MJ, et al (2009) A portrait of the "SCP/TAPS" proteins of eukaryotes - Developing a framework for fundamental research and biotechnological outcomes. Biotechnol Adv 27: 376–388. 19239923 10.1016/j.biotechadv.2009.02.005

[19] Cantacessi C, Gasser RB (2012) SCP/TAPS proteins in helminths - where to from now? Mol Cell Probes 26: 54–59. 22005034 10.1016/j.mcp.2011.10.001

[20] Jasmer DP, Goverse A, Smant G (2003) Parasitic Nematode Interactions with Mammals and Plants. Annu Rev Phytopathol 41: 245–270. 14527330 10.1146/annurev.phyto.41.052102.104023

[21] Hawdon JM, Jones BF, Hoffman DR, Hotez PJ (1996) Cloning and characterization of *Ancylostoma*-secreted protein: A novel protein associated with the transition to parasitism by infective hookworm larvae. J Biol Chem 271: 6672–6678. 8636085 10.1074/jbc.271.12.6672

[22] Hawdon JM, Narasimhan S, Hotez PJ (1999) *Ancylostoma* secreted protein 2: cloning and characterization of a second member of a family of nematode secreted proteins from *Ancylostoma caninum* . Mol Biochem Parasitol 99: 149–165. 10340481 10.1016/s0166-6851(99)00011-0

[23] Datu BJD, Gasser RB, Nagaraj SH, Ong EK, O'Donoghue P, et al (2008) Transcriptional changes in the hookworm, *Ancylostoma caninum*, during the transition from a free-living to a parasitic larva. PLoS Negl Trop Dis 2: e130. 18235850 10.1371/journal.pntd.0000130 PMC2217673

[24] Mulvenna J, Hamilton B, Nagaraj SH, Smyth D, Loukas A, et al (2009) Proteomics analysis of the excretory/secretory component of the blood-feeding stage of the hookworm, *Ancylostoma caninum* . Mol Cell Proteomics 8: 109–121. 18753127 10.1074/mcp.M800206-MCP200

[25] Haegeman A, Mantelin S, Jones JT, Gheysen G (2012) Functional roles of effectors of plant-parasitic nematodes. Gene 492: 19–31. 22062000 10.1016/j.gene.2011.10.040

[26] Hewezi T, Baum TJ (2013) Manipulation of plant cells by cyst and root-knot nematode effectors. Mol Plant Microbe Interact 26: 9–16. 22809272 10.1094/MPMI-05-12-0106-FI

[27] Mitchum MG, Hussey RS, Baum TJ, Wang X, Elling AA, et al (2013) Nematode effector proteins: An emerging paradigm of parasitism. New Phytol 199: 879–894. 23691972 10.1111/nph.12323

[28] Kyndt T, Vieira P, Gheysen G, de Almeida-Engler J (2013) Nematode feeding sites: unique organs in plant roots. Planta: 1–12. 10.1007/s00425-013-1923-z 23824525

[29] Chronis D, Chen SY, Lu SW, Hewezi T, Carpenter SCD, et al (2013) A ubiquitin carboxyl extension protein secreted from a plant-parasitic nematode *Globodera rostochiensis* is cleaved in planta to promote plant parasitism. Plant J 74: 185–196. 23346875 10.1111/tpj.12125

[30] Postma WJ, Slootweg EJ, Rehman S, Finkers-Tomczak A, Tytgat TOG, et al (2012) The effector SPRYSEC-19 of *Globodera rostochiensis* suppresses CC-NB-LRR-mediated disease resistance in plants. Plant Physiol 160: 944–954. 22904163 10.1104/pp.112.200188 PMC3461567

[31] Jaouannet M, Magliano M, Arguel MJ, Gourgues M, Evangelisti E, et al (2013) The root-knot nematode calreticulin Mi-CRT is a key effector in plant defense suppression. Mol Plant Microbe Interact 26: 97–105. 22857385 10.1094/MPMI-05-12-0130-R

[32] Jaubert S, Milac AL, Petrescu AJ, De Almeida-Engler J, Abad P, et al (2005) In planta secretion of a calreticulin by migratory and sedentary stages of root-knot nematode. Mol Plant Microbe Interact 18: 1277–1284. 16478047 10.1094/MPMI-18-1277

[33] Carpita N, McCann M, Griffing LR (1996) The plant extracellular matrix: News from the cell's frontier. Plant Cell 8: 1451–1463. 8837501 10.1105/tpc.8.9.1451 PMC161290

[34] Aumailley M, Gayraud B (1998) Structure and biological activity of the extracellular matrix. J Mol Med 76: 253–265. 9535559 10.1007/s001090050215

[35] Nurnberger T, Brunner F, Kemmerling B, Piater L (2004) Innate immunity in plants and animals: Striking similarities and obvious differences. Immunol Rev 198: 249–266. 15199967 10.1111/j.0105-2896.2004.0119.x

[36] Ausubel FM (2005) Are innate immune signaling pathways in plants and animals conserved? Nat Immunol 6: 973–979. 16177805 10.1038/ni1253

[37] Moyle M, Foster DL, McGrath DE, Brown SM, Laroche Y, et al (1994) A hookworm glycoprotein that inhibits neutrophil function is a ligand of the integrin CD11b/CD18. J Biol Chem 269: 10008–10015. 7908286

[38] Bower MA, Constant SL, Mendez S (2008) *Necator americanus:* The Na-ASP-2 protein secreted by the infective larvae induces neutrophil recruitment in vivo and in vitro. Exp Parasitol 118: 569–575. 18199436 10.1016/j.exppara.2007.11.014 PMC2359483

[39] Asojo OA, Goud G, Dhar K, Loukas A, Zhan B, et al (2005) X-ray structure of Na-ASP-2, a pathogenesis-related-1 protein from the nematode parasite, *Necator americanus*, and a vaccine antigen for human hookworm infection. J Mol Biol 346: 801–814. 15713464 10.1016/j.jmb.2004.12.023

[40] Del Valle A, Jones BF, Harrison LM, Chadderdon RC, Cappello M (2003) Isolation and molecular cloning of a secreted hookworm platelet inhibitor from adult *Ancylostoma caninum* . Mol Biochem Parasitol 129: 167–177. 12850261 10.1016/s0166-6851(03)00121-x

[41] Chinchilla D, Bauer Z, Regenass M, Boller T, Felix G (2006) The Arabidopsis receptor kinase FLS2 binds flg22 and determines the specificity of flagellin perception. Plant Cell 18: 465–476. 16377758 10.1105/tpc.105.036574 PMC1356552

[42] Van Esse HP, Van't Klooster JW, Bolton MD, Yadeta KA, Van Baarlen P, et al (2008) The *Cladosporium fulvum* virulence protein Avr2 inhibits host proteases required for basal defense. Plant Cell 20: 1948–1963. 18660430 10.1105/tpc.108.059394 PMC2518240

[43] Kaschani F, Shabab M, Bozkurt T, Shindo T, Schornack S, et al (2010) An effector-targeted protease contributes to defense against *Phytophthora infestans* and is under diversifying selection in natural hosts. Plant Physiol 154: 1794–1804. 20940351 10.1104/pp.110.158030 PMC2996022

[44] Ding X, Shields J, Allen R, Hussey RS (2000) Molecular cloning and characterisation of a venom allergen AG5-like cDNA from *Meloidogyne incognita* . Int J Parasitol 30: 77–81. 10675748 10.1016/s0020-7519(99)00165-4

[45] Gómez-Gómez L, Boller T (2000) FLS2: An LRR receptor-like kinase involved in the perception of the bacterial elicitor flagellin in Arabidopsis. Mol Cell 5: 1003–1011. 10911994 10.1016/s1097-2765(00)80265-8

[46] Kanehisa M, Goto S (2000) KEGG: Kyoto Encyclopedia of Genes and Genomes. Nucleic Acids Res 28: 27–30. 10592173 10.1093/nar/28.1.27 PMC102409

[47] Kanehisa M (2013) Molecular network analysis of diseases and drugs in KEGG. In: Mamitsuka H, Kanehisa M, DeLisi C, editors. pp.263–275. 10.1007/978-1-62703-107-3_17 23192552

[48] Kanehisa M, Goto S, Hattori M, Aoki-Kinoshita KF, Itoh M, et al (2006) From genomics to chemical genomics: new developments in KEGG. Nucleic Acids Res 34: D354–357. 16381885 10.1093/nar/gkj102 PMC1347464

[49] Schaller A, Stintzi A, Graff L (2012) Subtilases - versatile tools for protein turnover, plant development, and interactions with the environment. Physiol Plant 145: 52–66. 21988125 10.1111/j.1399-3054.2011.01529.x

[50] Ramírez V, López A, Mauch-Mani B, Gil MJ, Vera P (2013) An Extracellular Subtilase Switch for Immune Priming in Arabidopsis. PLoS Pathog 9: e1603445. 10.1371/journal.ppat.1003445 PMC3688555 23818851

[51] Li XP, Björkman O, Shih C, Grossman AR, Rosenquist M, et al (2000) A pigment-binding protein essential for regulation of photosynthetic light harvesting. Nature 403: 391–395. 10667783 10.1038/35000131

[52] Göhre V, Jones AME, Sklenár J, Robatzek S, Weber APM (2012) Molecular crosstalk between PAMP-triggered immunity and photosynthesis. Mol Plant Microbe Interact 25: 1083–1092. 22550958 10.1094/MPMI-11-11-0301

[53] Jankanpaa HJ, Frenkel M, Zulfugarov I, Reichelt M, Krieger-Liszkay A, et al (2013) Non-Photochemical Quenching Capacity in *Arabidopsis thaliana* Affects Herbivore Behaviour. PLoS One 8: e53232. 23301046 10.1371/journal.pone.0053232 PMC3534670

[54] Demmig-Adams B, Cohu CM, Amiard V, Zadelhoff G, Veldink GA, et al (2013) Emerging trade-offs - impact of photoprotectants (PsbS, xanthophylls, and vitamin E) on oxylipins as regulators of development and defense. New Phytol 197: 720–729. 23418633 10.1111/nph.12100

[55] Danon A, Miersch O, Felix G, den Camp RGLO, Apel K (2005) Concurrent activation of cell death-regulating signaling pathways by singlet oxygen in *Arabidopsis thaliana* . Plant J 41: 68–80. 15610350 10.1111/j.1365-313X.2004.02276.x

[56] Rehman S, Butterbach P, Popeijus H, Overmars H, Davis EL, et al (2009) Identification and characterization of the most abundant cellulases in stylet secretions from *Globodera rostochiensis* . Phytopathol 99: 194–202. 10.1094/PHYTO-99-2-0194 19245333

[57] Heil M (2012) Damaged-self recognition as a general strategy for injury detection. Plant Signaling and Behavior 7: 576–580. 22516811 10.4161/psb.19921 PMC3419023

[58] Sinha D, Gupta MK, Patel HK, Ranjan A, Sonti RV (2013) Cell Wall Degrading Enzyme Induced Rice Innate Immune Responses Are Suppressed by the Type 3 Secretion System Effectors XopN, XopQ, XopX and XopZ of *Xanthomonas oryzae* pv. *oryzae* . PLoS One 8: e75867. 24086651 10.1371/journal.pone.0075867 PMC3784402

[59] Bouwmeester K, Govers F (2009) Arabidopsis L-type lectin receptor kinases: Phylogeny, classification, and expression profiles. J Exp Bot 60: 4383–4396. 19773388 10.1093/jxb/erp277

[60] Nakhamchik A, Zhao Z, Provart NJ, Shiu SH, Keatley SK, et al (2004) A comprehensive expression analysis of the Arabidopsis proline-rich extensin-like receptor kinase gene family using bioinformatic and experimental approaches. Plant Cell Physiol 45: 1875–1881. 15653807 10.1093/pcp/pch206

[61] Silva NF, Goring DR (2002) The proline-rich, extensin-like receptor kinase-1 (PERK1) gene is rapidly induced by wounding. Plant Mol Biol 50: 667–685. 12374299 10.1023/a:1019951120788

[62] Shabab M, Shindo T, Gu C, Kaschani F, Pansuriya T, et al (2008) Fungal effector protein AVR2 targets diversifying defense-related cys proteases of tomato. Plant Cell 20: 1169–1183. 18451324 10.1105/tpc.107.056325 PMC2390736

[63] Rautengarten C, Steinhauser D, Bussis D, Stintzi A, Schaller A, et al (2005) Inferring hypotheses on functional relationships of genes: Analysis of the *Arabidopsis thaliana* subtilase gene family. PLoS Comput Biol 1: 297–312. 10.1371/journal.pcbi.0010040 PMC1236819 16193095

[64] Li XP, Gilmore AM, Caffarri S, Bassi R, Golan T, et al (2004) Regulation of photosynthetic light harvesting involves intrathylakoid lumen pH sensing by the PsbS protein. J Biol Chem 279: 22866–22874. 15033974 10.1074/jbc.M402461200

[65] Roach T, Krieger-Liszkay A (2012) The role of the PsbS protein in the protection of photosystems I and II against high light in *Arabidopsis thaliana* . BBA Bioenergetics 1817: 2158–2165. 23000078 10.1016/j.bbabio.2012.09.011

[66] Triantaphylides C, Havaux M (2009) Singlet oxygen in plants: production, detoxification and signaling. Trends Plant Sci 14: 219–228. 19303348 10.1016/j.tplants.2009.01.008

[67] Li XP, Muller-Moule P, Gilmore AM, Niyogi KK (2002) PsbS-dependent enhancement of feedback de-excitation protects photosystem II from photoinhibition. Proc Natl Acad Sci U S A 99: 15222–15227. 12417767 10.1073/pnas.232447699 PMC137571

[68] Frenkel M, Kulheim C, Jankanpaaa HJ, Skogstrom O, Dall'Osto L, et al (2009) Improper excess light energy dissipation in Arabidopsis results in a metabolic reprogramming. BMC Plant Biol 9. 10.1186/1471-2229-9-12 PMC2656510 19171025

[69] Golinowski W, Grundler FMW, Sobczak M (1996) Changes in the structure of *Arabidopsis thaliana* during female development of the plant-parasitic nematode *Heterodera schachtii* . Protoplasma 194: 103–116.

[70] Szakasits D, Heinen P, Wieczorek K, Hofmann J, Wagner F, et al (2009) The transcriptome of syncytia induced by the cyst nematode *Heterodera schachtii* in Arabidopsis roots. Plant J 57: 771–784. 18980640 10.1111/j.1365-313X.2008.03727.x PMC2667683

[71] Decreux A, Messiaen J (2005) Wall-associated kinase WAK1 interacts with cell wall pectins in a calcium-induced conformation. Plant Cell Physiol 46: 268–278. 15769808 10.1093/pcp/pci026

[72] Brutus A, Sicilia F, Macone A, Cervone F, De Lorenzo G (2010) A domain swap approach reveals a role of the plant wall-associated kinase 1 (WAK1) as a receptor of oligogalacturonides. Proc Natl Acad Sci U S A 107: 9452–9457. 20439716 10.1073/pnas.1000675107 PMC2889104

[73] D'Ovidio R, Mattei B, Roberti S, Bellincampi D (2004) Polygalacturonases, polygalacturonase-inhibiting proteins and pectic oligomers in plant-pathogen interactions. BBA Proteins Proteom 1696: 237–244. 10.1016/j.bbapap.2003.08.012 14871664

[74] Moerschbacher BM, Mierau M, Graeßner B, Noll U, Mort AJ (1999) Small oligomers of galacturonic acid are endogenous suppressors of disease resistance reactions in wheat leaves. J Exp Bot 50: 605–612.

[75] Chen Q, Rehman S, Smant G, Jones JT (2005) Functional analysis of pathogenicity proteins of the potato cyst nematode *Globodera rostochiensis* using RNAi. Mol Plant Microbe Interact 18: 621. 16042007 10.1094/MPMI-18-0621

[76] Greenbaum D, Medzihradszky KF, Burlingame A, Bogyo M (2000) Epoxide electrophiles as activity-dependent cysteine protease profiling and discovery tools. Chem Biol 7: 569–581. 11048948 10.1016/s1074-5521(00)00014-4

[77] Curtis MD, Grossniklaus U (2003) A Gateway Cloning Vector Set for High-Throughput Functional Analysis of Genes *in Planta* . Plant Physiol 133: 462–469. 14555774 10.1104/pp.103.027979 PMC523872

[78] De Boer JM, Overmars HA, Bakker J, Gommers FJ (1992) Analysis of two-dimensional protein patterns from developmental stages of the potato cyst-nematode, *Globodera rostochiensis* . Parasitology 105: 461–474.

[79] Martin J, Abubucker S, Heizer E, Taylor CM, Mitreva M (2012) Nematode.net update 2011: Addition of data sets and tools featuring next-generation sequencing data. Nucleic Acids Res 40: D720–D728. 22139919 10.1093/nar/gkr1194 PMC3245159

[80] Nakagawa T, Suzuki T, Murata S, Nakamura S, Hino T, et al (2007) Improved gateway binary vectors: High-performance vectors for creation of fusion constructs in transgenic analysis of plants. Biosci Biotech Biochem 71: 2095–2100. 10.1271/bbb.70216 17690442

[81] Clough SJ, Bent AF (1998) Floral dip: A simplified method for *Agrobacterium*-mediated transformation of *Arabidopsis thaliana* . Plant J 16: 735–743. 10069079 10.1046/j.1365-313x.1998.00343.x

[82] Sijmons PC, Grundler FMW, Von Mende N, Burrows PR, Wyss U (1991) *Arabidopsis thaliana* as a new model host for plant-parasitic nematodes. Plant J 1: 245–254.

[83] Baum TJ, Wubben MTE Ii, Hardy KA, Su H, Rodermel SR (2000) A screen for *Arabidopsis thaliana* mutants with altered susceptibility to *Heterodera schachtii* . J Nematol 32: 166–173. 19270962 PMC2620447

[84] Van Esse HP, Bolton MD, Stergiopoulos I, De Wit PJGM, Thomma BPHJ (2007) The chitin-binding *Cladosporium fulvum* effector protein Avr4 is a virulence factor. Mol Plant Microbe Interact 20: 1092–1101. 17849712 10.1094/MPMI-20-9-1092

[85] Bouwmeester K, de Sain M, Weide R, Gouget A, Klamer S, et al (2011) The lectin receptor kinase LecRK-I.9 is a novel Phytophthora resistance component and a potential host target for a RXLR effector. PLoS Pathog 7: e1001327. 21483488 10.1371/journal.ppat.1001327 PMC3068997

[86] van de Mortel JE, de Vos RCH, Dekkers E, Pineda A, Guillod L, et al (2012) Metabolic and transcriptomic changes induced in arabidopsis by the rhizobacterium Pseudomonas fluorescens SS101. Plant Physiol 160: 2173–2188. 23073694 10.1104/pp.112.207324 PMC3510139

[87] Pieterse CMJ, Van Wees SCM, Hoffland E, Van Pelt JA, Van Loon LC (1996) Systemic resistance in Arabidopsis induced by biocontrol bacteria is independent of salicylic acid accumulation and pathogenesis-related gene expression. Plant Cell 8: 1225–1237. 8776893 10.1105/tpc.8.8.1225 PMC161233

[88] Gómez-Gómez L, Felix G, Boller T (1999) A single locus determines sensitivity to bacterial flagellin in *Arabidopsis thaliana* . Plant J 18: 277–284. 10377993 10.1046/j.1365-313x.1999.00451.x

[89] Robinson MD, McCarthy DJ, Smyth GK (2010) edgeR: a Bioconductor package for differential expression analysis of digital gene expression data. Bioinformatics 26: 139–140. 19910308 10.1093/bioinformatics/btp616 PMC2796818

[90] Tanz SK, Castleden I, Hooper CM, Vacher M, Small I, et al (2013) SUBA3: A database for integrating experimentation and prediction to define the SUBcellular location of proteins in Arabidopsis. Nucleic Acids Res 41: D1185–D1191. 23180787 10.1093/nar/gks1151 PMC3531127

