# Supplementary figures and images for: Apoplastic Venom Allergen-like Proteins of Cyst Nematodes Modulate the Activation of Basal Plant Innate Immunity by Cell Surface Receptors
Source: PLoS Pathog. 2014 Dec 11;10(12):e1004569. doi: 10.1371/journal.ppat.1004569 (PMC4263768; doi:10.1371/journal.ppat.1004569)

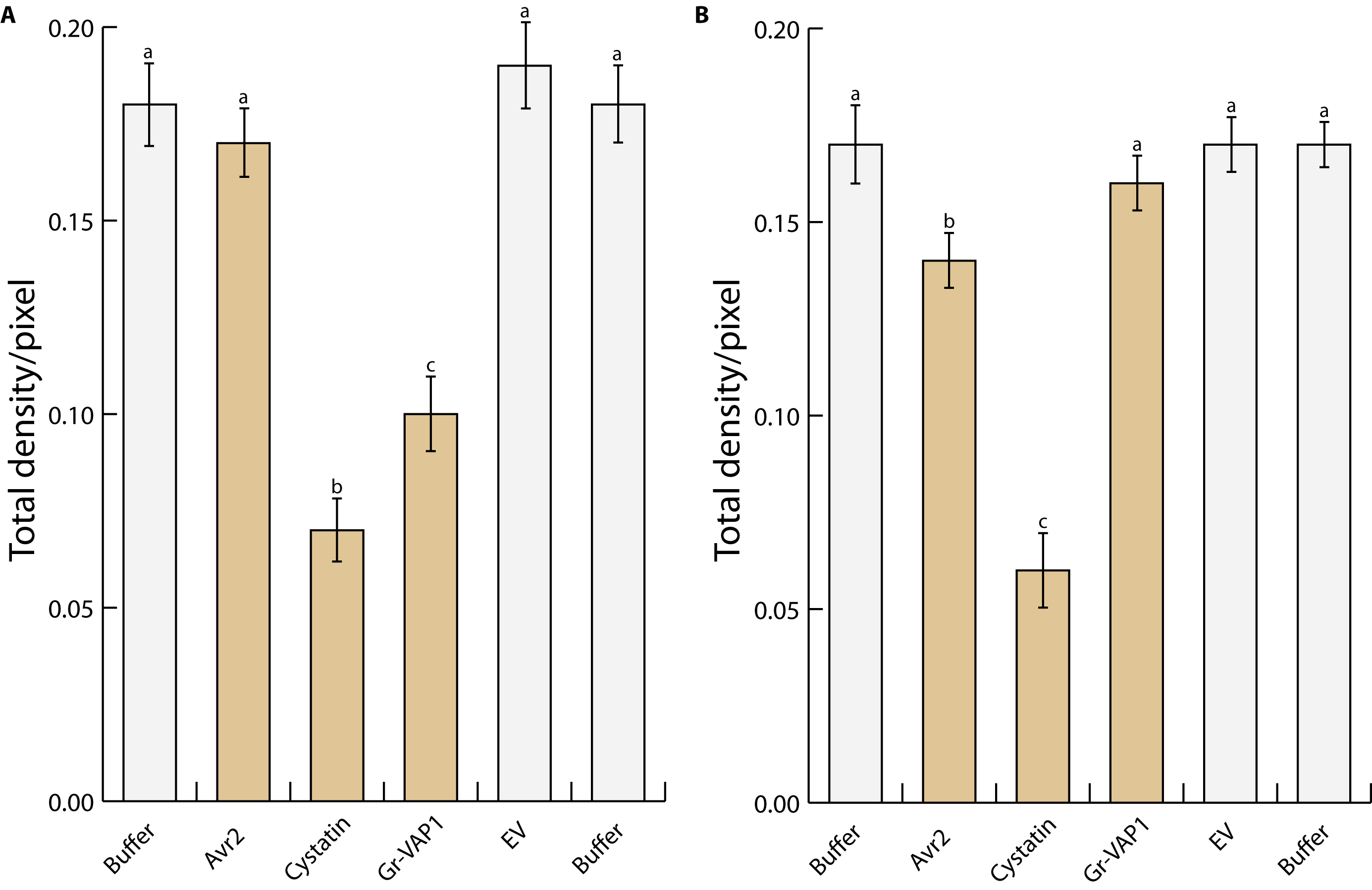

Supplement: S1 Figure — Apoplastic Gr-VAP1 perturbs the active site of the extracellular defense-related papain-like cysteine protease C14tub of potato ( Solanum tuberosum ). Labeling densities of the fluorescent activity-based probe DCG-04 to the active site of (A) C14tub and (B) C14lyc of tomato (S. lycopersicum) following treatment with Gr-VAP1 isolated from apoplastic fluids of agroinfiltrated leaves. Treatments with the Avr2, egg white cystatin, and apoplastic fluids from agroinfiltrations with the empty binary expression vector (EV), and with buffer alone (Buffer) were included as controls. Labeling densities were quantified in triplicates and statistical significance of differences was determined with an ANOVA. Different letters indicate significant differences when using of P-value <0.05 as threshold. (TIF) [file ppat.1004569.s001.tif]

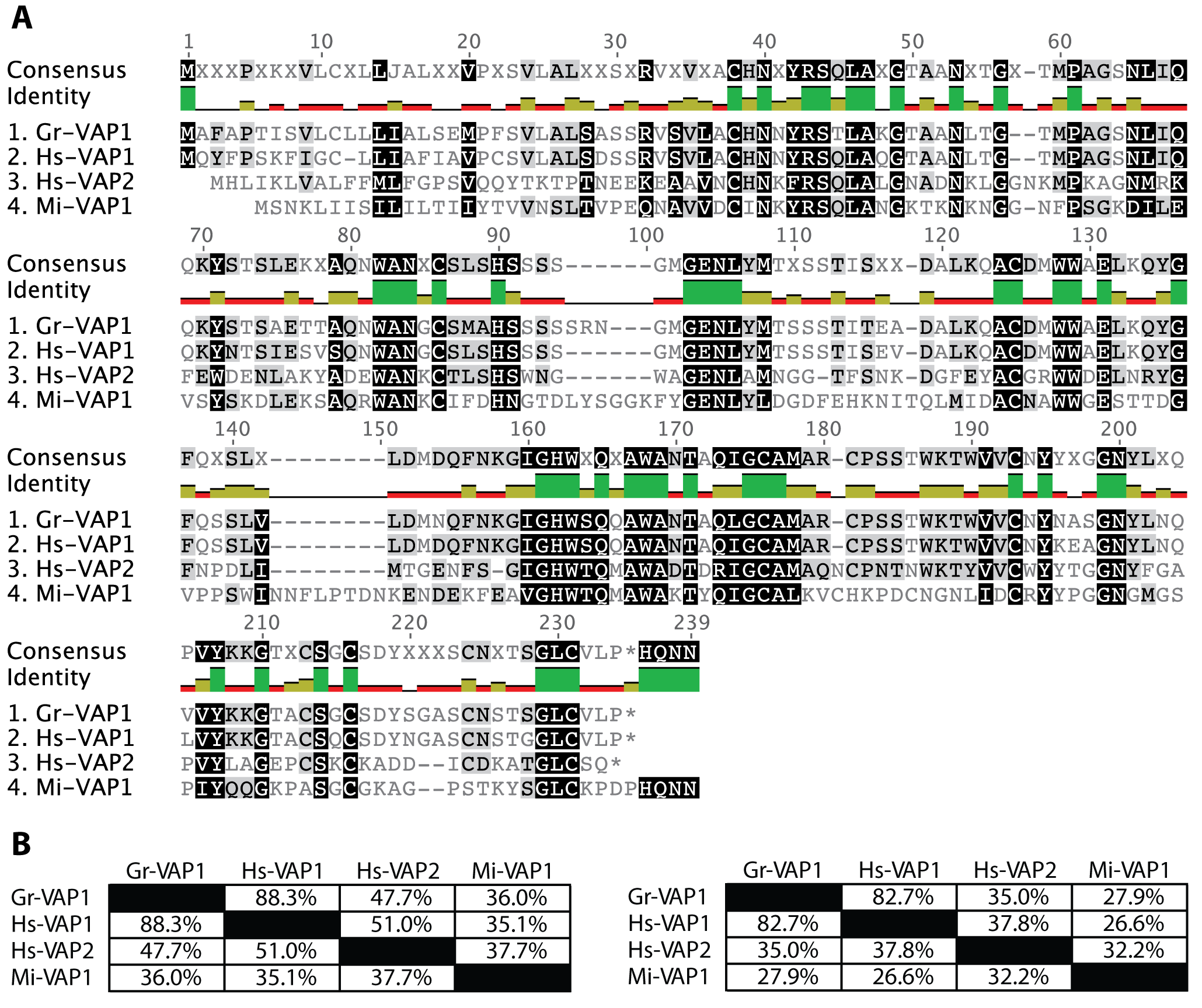

Supplement: S2 Figure — Protein sequence variation in venom allergen-like proteins from cyst nematodes and root-knot nematodes. (A) Protein sequence alignment of venom allergen-like proteins from the potato cyst nematode Globodera rostochiensis (Gr-VAP1), the beet cyst nematode Heterodera schachtii (Hs-VAP1 and Hs-VAP2), and the root-knot nematode Meloidogyne incognita (Mi-VAP1). Colors indicate identity (black background) or similarity among the sequences (gray background). (B) Protein similarity matrix of venom allergen-like proteins. Numbers represent the percentage of amino acid residues that are similar (bottom left corner) and identical for any pair of proteins. (TIF) [file ppat.1004569.s002.tif]

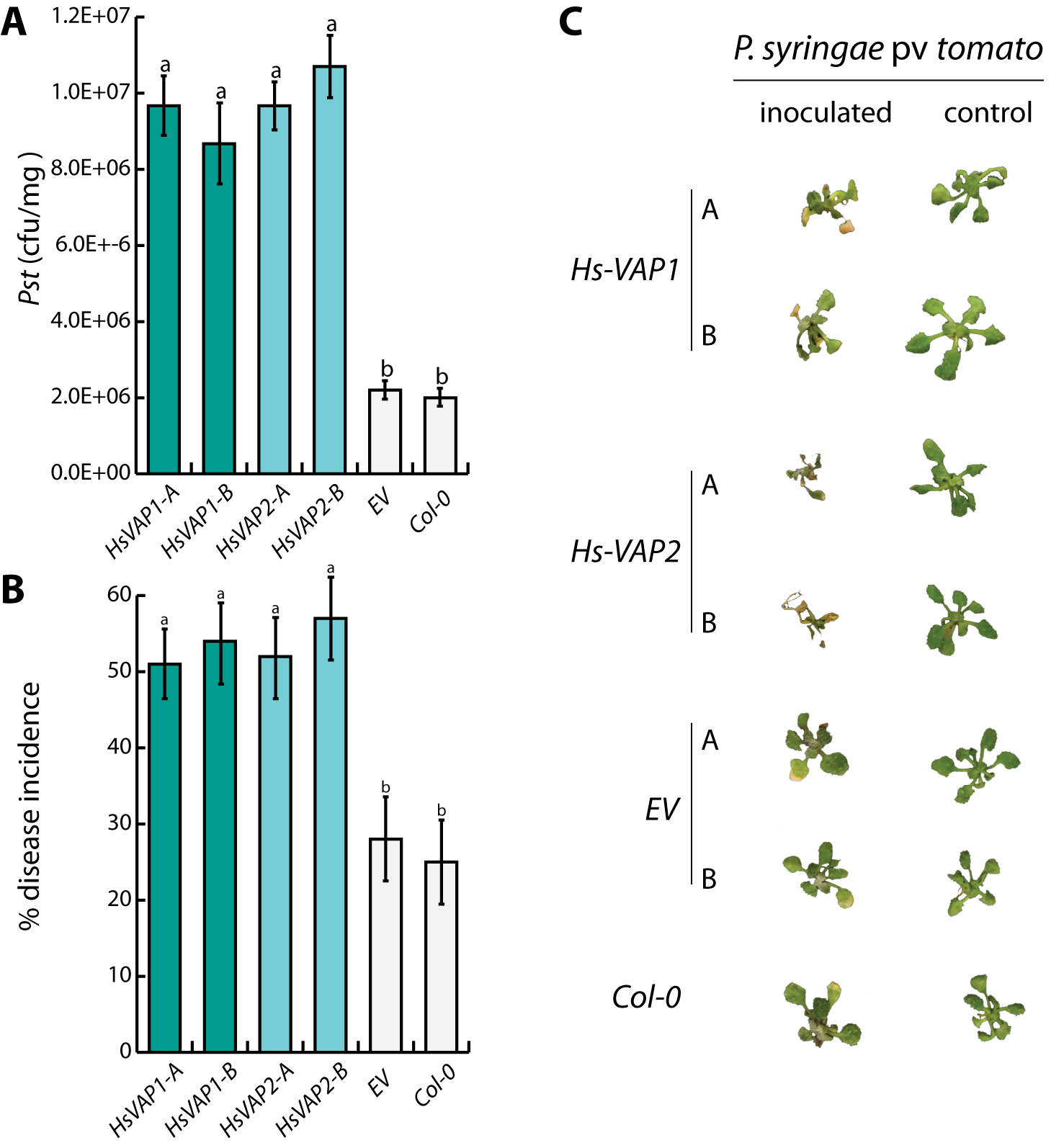

Supplement: S3 Figure — Ectopic venom allergen-like proteins enhance susceptibility to Pseudomonas syringae pv. tomato ( Pst ) in Arabidopsis. Heterologous expression of the venom allergen-like proteins Hs-VAP1 and Hs-VAP2 from Heterodera schachtii in the apoplast of transgenic Arabidopsis lines enhances their susceptibility to Pst DC3000. Two independent transgenic lines per construct (-A and -B) were compared with corresponding transgenic line harboring the T-DNA of the empty vector (EV) and wild type A. thaliana (Col-0). (A) Population densities were determined 4 days after inoculation with Pst. Bars represent colony forming units (cfu/mg of tissue) for three independent replicates of 8 plants each. (B) Disease incidence was evaluated 4 days after inoculation. Bars represent the mean percentage of leaves, which had developed chlorotic symptoms of 24 plants. Statistical significance of differences was determined with an ANOVA. Different letters indicate statistical significance when using P-value <0.05 as threshold. (C) Pictures show typical symptoms on Arabidopsis plants inoculated either with Pst, or mock inoculated. (TIF) [file ppat.1004569.s003.tif]

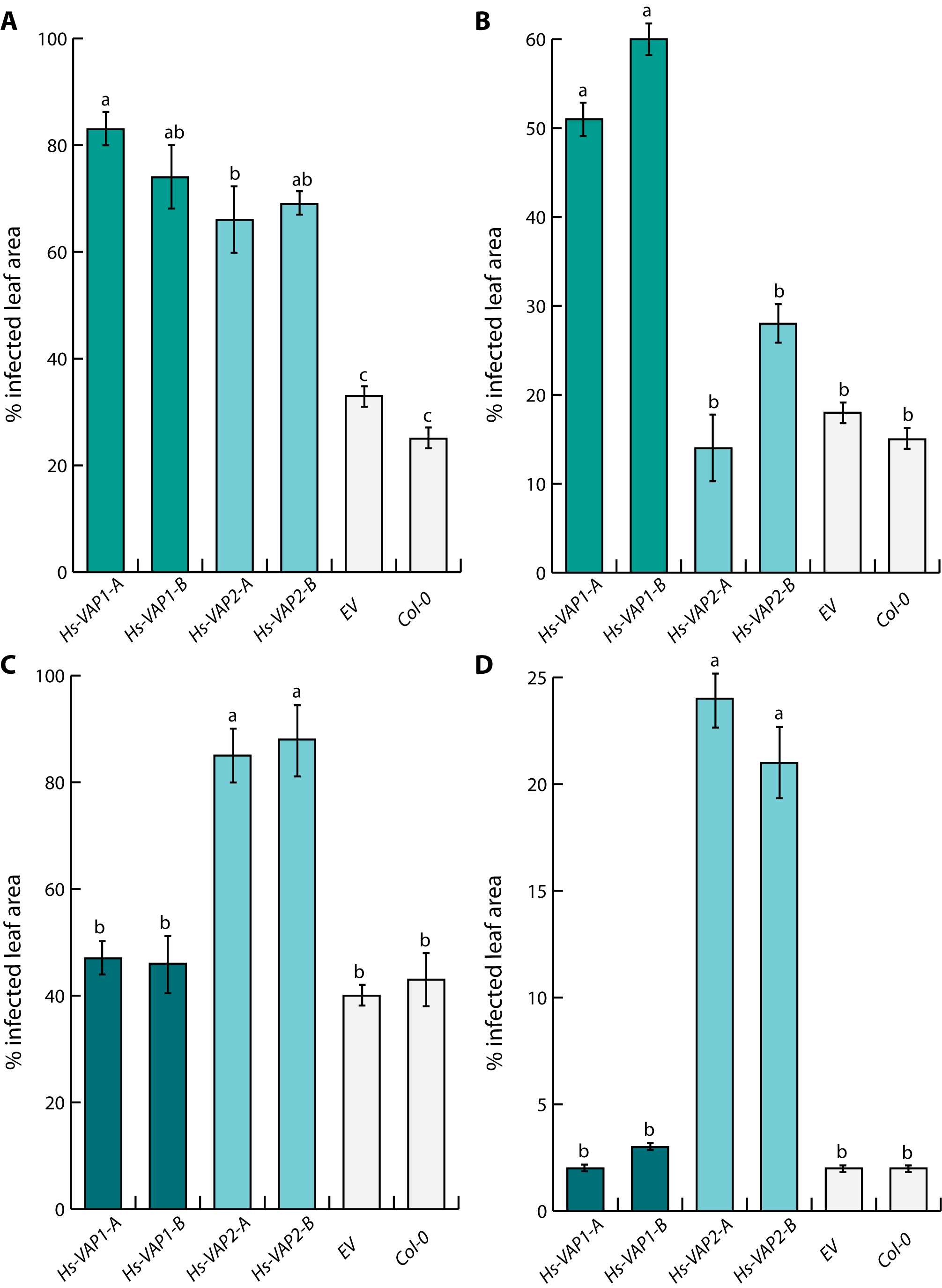

Supplement: S4 Figure — Ectopic venom allergen-like proteins enhance development of disease symptoms of fungal and oomycete pathogens in Arabidopsis. Bars represent mean percentage infected leaf area of transgenic Arabidopsis line overexpressing Hs-VAP1 and Hs-VAP2 3 days after inoculation with (A) Botrytis cinerea, (B) Plectosphaerella cucumerina, and (C and D) two isolates of Phytophthora brassicae (CBS686.95 and HH). Statistical significance of differences with transgenic plants harboring the T-DNA of the corresponding empty expression vector (EV) and wild type Arabidopsis (Col-0) was determined with an ANOVA. Different letters indicate significant differences when using of P-value <0.05 as threshold. (TIF) [file ppat.1004569.s004.tif]

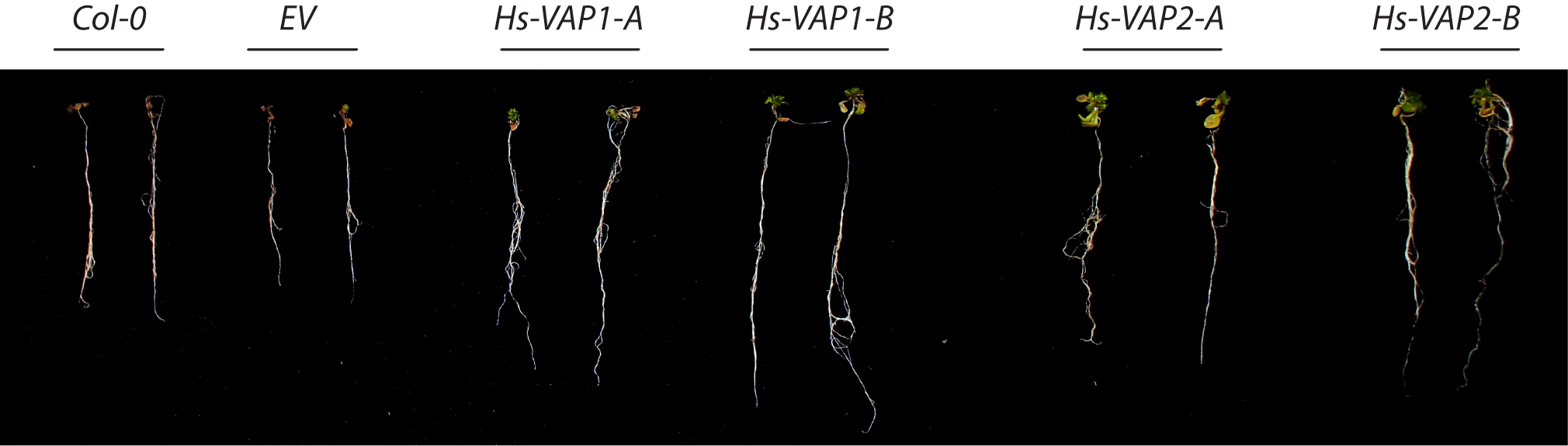

Supplement: S5 Figure — Ectopic venom allergen-like proteins abrogate the inhibition of seedling growth by flg22 in Arabidopsis. Photograph of typical root length of transgenic Arabidopsis lines overexpressing Hs-VAP1 and Hs-VAP2 after 10 days of growth in the presence of 10 µM flg22. Transgenic plants harboring the T-DNA of the corresponding empty expression vector (EV) and wild type Arabidopsis (Col-0) were used to show the normal inhibition of root growth in the presence of flg22. (TIF) [file ppat.1004569.s005.tif]

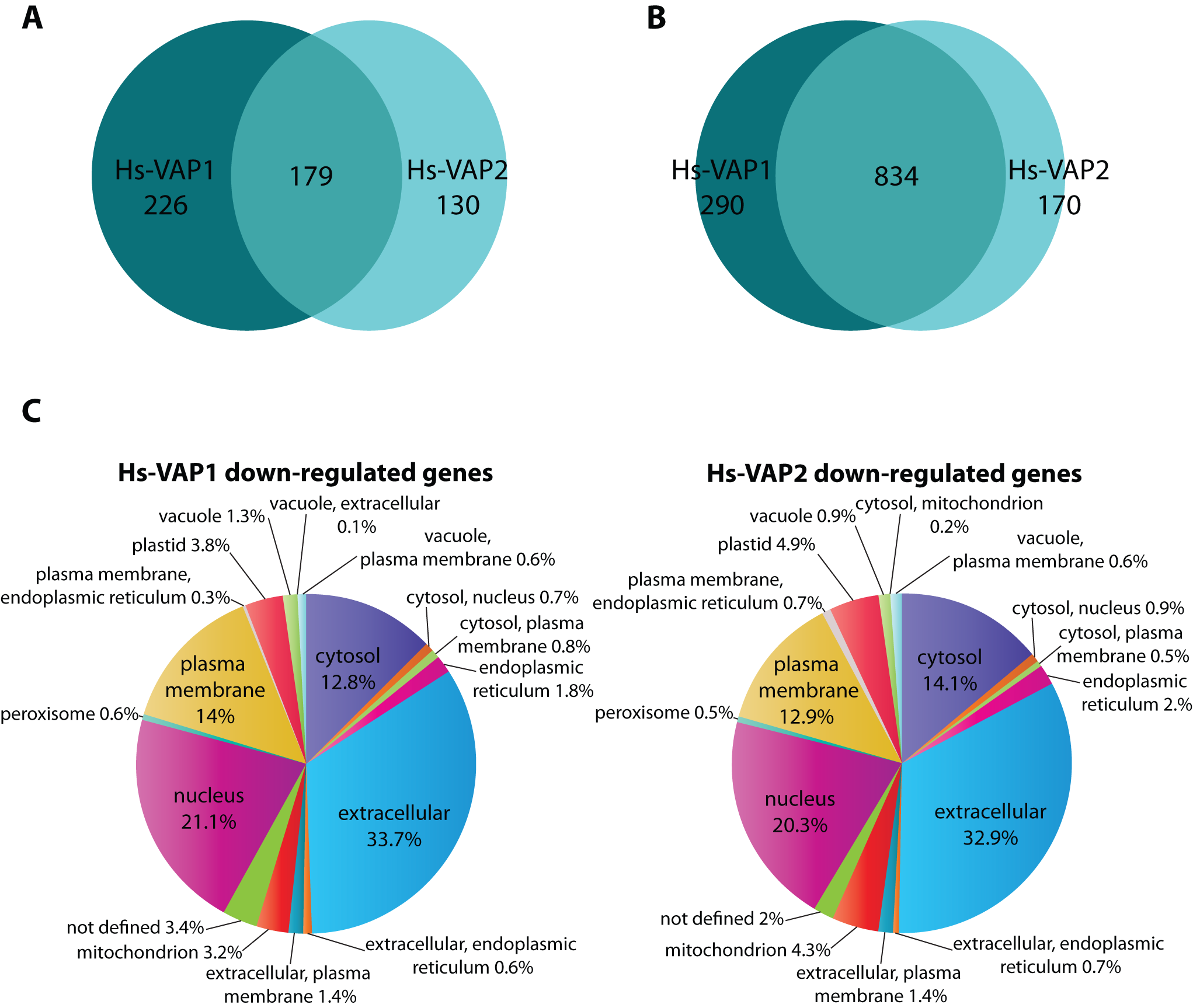

Supplement: S6 Figure — Ectopic venom allergen-like proteins regulate immunity related pathways in Arabidopsis. Global gene expression analysis as determined by RNA-seq in 2 weeks old Arabidopsis plants overexpressing Hs-VAP1 and Hs-VAP2 in the apoplast. Venn's diagrams depict the total number of significantly up- (A) and down-regulated (B) genes relative to transgenic Arabidopsis plants harboring T-DNA of the corresponding empty expression vector (EV) when using a false discovery rate of 0.05 as cutoff. (C) Pie charts depict percentage of products of genes significantly down-regulated by ectopic Hs-VAP1 and Hs-VAP2 in Arabidopsis, according to their predicted subcellular localization in the SUBA database. (TIF) [file ppat.1004569.s006.tif]

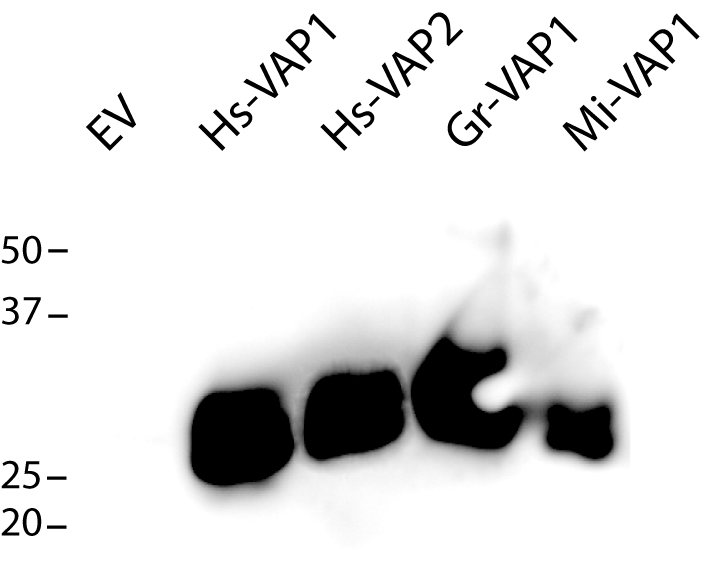

Supplement: S7 Figure — VAPs harboring their native signal peptide for secretion are secreted to the apoplast of agroinfiltrated leaves of Nicotiana benthamiana . Heterodera schachtii VAPs (Hs-VAP1 and –VAP2), Globodera rostochiensis VAP1 (Gr-VAP1), and Meloidogyne incognita VAP1 (Mi-VAP1) were transiently expressed in N. benthamiana plants as recombinant carboxyl terminus FLAG tagged proteins together with empty vector (EV) controls. VAPs were detected in apoplastic fluids isolated from agroinfiltrated leaf segments at 5 days post infiltration on western blots using FLAG specific antibody. (TIF) [file ppat.1004569.s007.tif]
