# Supplementary material for: Apoplastic Venom Allergen-like Proteins of Cyst Nematodes Modulate the Activation of Basal Plant Innate Immunity by Cell Surface Receptors
Source: PLoS Pathog. 2014 Dec 11;10(12):e1004569. doi: 10.1371/journal.ppat.1004569 (PMC4263768; doi:10.1371/journal.ppat.1004569)
Supplement: S5 Table — KEGG pathway enrichment analysis: Statistically enriched pathways among differentially expressed genes in Arabidopsis thaliana overexpressing Hs-VAP1 and Hs-VAP2 relative to the corresponding transgenic empty vector plants. (DOCX) [file ppat.1004569.s012.docx]

| **Table S5.** KEGG pathway enrichment analysis: statistically enriched pathways among differentially expressed genes in *Arabidopsis thaliana* overexpressing *Hs-VAP1* and *Hs-VAP2* relative to the corresponding transgenic empty vector plants. | | | | | | | | | |
| --- | --- | --- | --- | --- | --- | --- | --- | --- | --- |
|  |  |  | | **Hs-VAP1** | | | **Hs-VAP2** | | |
| **Pathway ID** | **Pathway term** | | **Rank** | | ***P*-value** | **Q-value** | **Rank** | ***P*-Value** | **Q-value** |
| KO04626 | Plant-pathogen interaction | | 1 | | 6.71E-15 | 8.59E-13 | 1 | 1.79E-14 | 2.30E-12 |
| KO02010 | ABC transporters | | 2 | | 8.28E-07 | 5.30E-05 | 2 | 3.51E-07 | 2.25E-05 |
| KO00402 | Benzoxazinoid biosynthesis | | 3 | | 2.47E-05 | 1.06E-03 | 3 | 4.65E-06 | 1.98E-04 |
| KO00190 | Oxidative phosphorylation | | 4 | | 7.83E-05 | 0.0025 | 4 | 7.22E-05 | 0.0023 |
| KO00941 | Flavonoid biosynthesis | | 5 | | 0.0003 | 0.0065 | 6 | 0.0007 | 0.0142 |
| KO03010 | Ribosome | | 6 | | 0.0003 | 0.0073 | 5 | 0.0002 | 0.0056 |
| KO04075 | Plant hormone signal transduction | | 7 | | 0.0011 | 0.0199 | 9 | 0.0048 | 0.0682 |
| KO04145 | Phagosome | | 8 | | 0.0013 | 0.021502 | 7 | 0.0021 | 0.0378 |
| KO00520 | Amino sugar and nucleotide sugar metabolism | | 9 | | 0.0042 | 0.0499 | 10 | 0.0058 | 0.0744 |
| KO00195 | Photosynthesis | | 10 | | 0.0042 | 0.0499 | 8 | 0.0041 | 0.0650 |
| The enrichment *P*-value was calculated using ultra-geometric test and corrected for multiple hypothesis testing by calculating Q-values. | | | | | | | | | |
