# Supplementary material for: Apoplastic Venom Allergen-like Proteins of Cyst Nematodes Modulate the Activation of Basal Plant Innate Immunity by Cell Surface Receptors
Source: PLoS Pathog. 2014 Dec 11;10(12):e1004569. doi: 10.1371/journal.ppat.1004569 (PMC4263768; doi:10.1371/journal.ppat.1004569)
Supplement: S6 Table — Plant immune receptors and cognate pathogen elicitors used to induce programmed cell death in leaves of Nicotiana benthamiana. (DOCX) [file ppat.1004569.s013.docx]

| **Table S6.** Plant immune receptors and cognate pathogen elicitors used to induce programmed cell death in leaves of *Nicotiana benthamiana*. | | | | |
| --- | --- | --- | --- | --- |
| **Immune receptor** | **Type*** | **Origin of receptor** | **Effector** | **Origin of cognate elicitor** |
| - | - | - | Inf1 | *Phytophthora infestans* |
| Cf4 | *RLP* | *Solanum hirsutum* | Avr4 | *Cladosporium fulvum* |
| Cf9 | *RLP* | *Solanum pimpinellifolium* | Avr9 | *Cladosporium fulvum* |
| R3a | *CC-NB-LRR* | *Solanum demissum* | Avr3a | *Phytophthora infestans* |
| Rpiblb1 | *CC-NB-LRR* | *Solanum bulbocastanum* | Avr-blb1 | *Phytophthora infestans* |
| Rpiblb2 | *CC-NB-LRR* | *Solanum bulbocastanum* | Avr-blb2 | *Phytophthora infestans* |
| Rx1 | *CC-NB-LRR* | *Solanum tuberosum* | CP | *Potato Virus X* |
| **RLP, transmembrane receptor-like proteins; CC-NB-LRR, intracellular coiled-coil nucleotide binding leucine-rich repeat proteins* | | | | |
