# Supplementary material for: Apoplastic Venom Allergen-like Proteins of Cyst Nematodes Modulate the Activation of Basal Plant Innate Immunity by Cell Surface Receptors
Source: PLoS Pathog. 2014 Dec 11;10(12):e1004569. doi: 10.1371/journal.ppat.1004569 (PMC4263768; doi:10.1371/journal.ppat.1004569)
Supplement: S7 Table — Oligonucleotide primers used in this study. (DOCX) [file ppat.1004569.s014.docx]

| **Table S7.** Oligonucleotide primers used in this study. | |
| --- | --- |
| **Name** | **Oligonucleotide sequence (5’- to 3’-end)** |
| Gr-VAP1-RNAiFw | CCCGGGTGCCACAACAACTACCGCTC |
| Gr-VAP1-RNAiR | TAATACGACTCACTATAGGGGGGTCATTAGTGAATATACCG |
| Gr-VAP1-sRTFw | TGCCACAACAACTACCGCTC |
| Gr-VAP1-sRTRv | AAGCCGAAAGAATAATTTATC |
| 60S-RTFw | GTGAAATCCGCAAACAACTG |
| 60S-RTRv | AGAGCCTGGAAGAACGAC |
| Gr-VAP1-RTFw | GCATTGGGCATTGGAGTC |
| Gr-VAP1-RTRv | TTTGTAGACGACCTGGTTC |
| cAMP-RTFw | ATCAGCCCATTCAAATCTACG |
| cAMP-RTRv | TTCTTCAGCAAGTCCTTCAAC |
| Gr-VAP1-GWFw | CACCATGGCGTTTGCCCCAACAAT |
| Gr-VAP1-GWRv | TGGCAAAACGCACAGTCCGCTGGT |
| StActinF | GCTTCCCGATGGTCAAGTCA |
| StActinR | GGATTCCAGCTGCTTCCATTC |
| qGrVAP1-Fw | AGCAGTACGGGTTTCAATCG |
| qGrVAP1-Rv | AACCCAAGTCTTCCATGTCG |
| Hs-VAP1-GWFw | CACCATGCAATATTTTCCATCTAAAT |
| HsVAP1-GwRv | TCATGGCAATACGCACAGTCCGCCGG |
| qHsVAP1-F | ACGAGCATTGAGTCGGTTTC |
| qHsVAP1-R | TGTTTCAGCGCATCAACTTC |
| HsVAP2-GWFw | CACCATGCATTTGATTAAATTAGT |
| HsVAP2-GwRv | TCATTGAGAGCAAAGTCCCGTGGC |
| qHsVAP2-F | GCGAGAATTTGGCAATGAAT |
| qHsVAP2-R | TTTTCCCCGGTCATAATCAG |
| AtClathrinF | GTTTGGGAGAAGAGCGGTTA |
| AtClathrinR | CTGATGTCACTGAACCTGAACTG |
